# Global Burden of Sickle Cell Anaemia in Children under Five, 2010–2050: Modelling Based on Demographics, Excess Mortality, and Interventions

**DOI:** 10.1371/journal.pmed.1001484

**Published:** 2013-07-16

**Authors:** Frédéric B. Piel, Simon I. Hay, Sunetra Gupta, David J. Weatherall, Thomas N. Williams

**Affiliations:** 1 Evolutionary Ecology of Infectious Disease, Department of Zoology, University of Oxford, Oxford, United Kingdom; 2 Spatial Ecology and Epidemiology Group, Department of Zoology, University of Oxford, Oxford, United Kingdom; 3 Global Network for Sickle Cell Disease, Toronto, Ontario, Canada; 4 Weatherall Institute of Molecular Medicine, University of Oxford, Oxford, United Kingdom; 5 Kenya Medical Research Institute/Wellcome Trust Programme, Centre for Geographic Medicine Research-Coast, Kilifi District Hospital, Kilifi, Kenya; 6 Department of Medicine, Imperial College, St Mary's Hospital, London, United Kingdom; Institute for Global Health, United Kingdom

## Abstract

**Background:**

The global burden of sickle cell anaemia (SCA) is set to rise as a consequence of improved survival in high-prevalence low- and middle-income countries and population migration to higher-income countries. The host of quantitative evidence documenting these changes has not been assembled at the global level. The purpose of this study is to estimate trends in the future number of newborns with SCA and the number of lives that could be saved in under-five children with SCA by the implementation of different levels of health interventions.

**Methods and Findings:**

First, we calculated projected numbers of newborns with SCA for each 5-y interval between 2010 and 2050 by combining estimates of national SCA frequencies with projected demographic data. We then accounted for under-five mortality (U5m) projections and tested different levels of excess mortality for children with SCA, reflecting the benefits of implementing specific health interventions for under-five patients in 2015, to assess the number of lives that could be saved with appropriate health care services. The estimated number of newborns with SCA globally will increase from 305,800 (confidence interval [CI]: 238,400–398,800) in 2010 to 404,200 (CI: 242,500–657,600) in 2050. It is likely that Nigeria (2010: 91,000 newborns with SCA [CI: 77,900–106,100]; 2050: 140,800 [CI: 95,500–200,600]) and the Democratic Republic of the Congo (2010: 39,700 [CI: 32,600–48,800]; 2050: 44,700 [CI: 27,100–70,500]) will remain the countries most in need of policies for the prevention and management of SCA. We predict a decrease in the annual number of newborns with SCA in India (2010: 44,400 [CI: 33,700–59,100]; 2050: 33,900 [CI: 15,900–64,700]). The implementation of basic health interventions (e.g., prenatal diagnosis, penicillin prophylaxis, and vaccination) for SCA in 2015, leading to significant reductions in excess mortality among under-five children with SCA, could, by 2050, prolong the lives of 5,302,900 [CI: 3,174,800–6,699,100] newborns with SCA. Similarly, large-scale universal screening could save the lives of up to 9,806,000 (CI: 6,745,800–14,232,700) newborns with SCA globally, 85% (CI: 81%–88%) of whom will be born in sub-Saharan Africa. The study findings are limited by the uncertainty in the estimates and the assumptions around mortality reductions associated with interventions.

**Conclusions:**

Our quantitative approach confirms that the global burden of SCA is increasing, and highlights the need to develop specific national policies for appropriate public health planning, particularly in low- and middle-income countries. Further empirical collaborative epidemiological studies are vital to assess current and future health care needs, especially in Nigeria, the Democratic Republic of the Congo, and India.

*Please see later in the article for the Editors' Summary*

## Abstract 2

Frédéric Piel and colleagues combine national sickle cell anemia (SCA) frequencies with projected demographic data to estimate the number of SCA births in children under five globally from 2010 to 2050, and then estimate the number of lives that could be be saved following implementation of specific health interventions starting in 2015.

*Please see later in the article for the Editors' Summary*

## Editors' Summary

**Background:**

More than seven million babies are born each year with a structural or functional abnormality. Although some birth defects are caused by environmental factors, many are caused by the inheritance of a defective gene. One common inherited birth defect is sickle cell anemia (SCA). SCA arises when a baby inherits the gene for sickle hemoglobin (HbS), a structural variant of normal adult hemoglobin (HbA, the protein in the disc-shaped red blood cells that carry oxygen round the body), from both its parents. Every cell in the human body contains two full sets of genes, and babies inherit one set of genes from each parent. The parents usually each have one HbS gene and one HbA gene, and are unaffected. However, the red blood cells of their offspring who inherit two copies of HbS develop a sickle (crescent) shape. Sickle cells can block blood vessels in the limbs and organs and have a shorter lifespan than normal red blood cells, which causes anemia. Together, these changes can cause acute pain and organ damage, and can increase the risk of severe infections. SCA can be prevented by prenatal diagnosis and managed by interventions such as the provision of antibiotics and vaccination to prevent infections.

**Why Was This Study Done?:**

Without early diagnosis and treatment, children with SCA often die within the first few years of life. Having one copy of the HbS gene provides people with protection from malaria, therefore SCA occurs mainly in low- and middle-income countries in tropical regions, where early diagnosis and treatment is often unavailable. Recent improvements in overall infant and childhood survival in these countries and population migration to higher-income countries mean that the global burden of SCA is likely to increase over the coming decades. To date, no one has tried to quantify this increase, although this information is needed to guide decisions on public health spending. In this modeling study, the researchers assess the size of the expected global burden of SCA between 2010 and 2050 in children under five years old and estimate the number of newborn lives that might be saved by implementation of various health interventions.

**What Did the Researchers Do and Find?:**

The researchers used estimates of national SCA frequencies and data on projected birth rates to calculate that the number of newborns with SCA will increase from about 305,800 in 2010 to about 404,200 in 2050. They estimated that Nigeria, the Democratic Republic of Congo (DRC), and India accounted for 57% of newborns with SCA in 2010, and that Nigeria and the DRC will probably still be the countries most in need of policies for the prevention and management of SCA in 2050. The researchers then assessed how many newborns might be saved by the implementation of various health measures in 2015 that affect excess mortality (the difference between the frequency of SCA in newborns and in five-year-olds divided by the frequency of SCA in newborns) among children born with SCA. Implementation of prenatal diagnosis and newborn screening programs, and provision of antibiotics and vaccinations (interventions assumed by the researchers to reduce excess mortality from 90% to 50% in low- and middle-income countries and from 10% to 5% in high-income countries) could prolong the life of more than five million newborns with SCA by 2050. Implementation of universal screening and provision of other specific measures predicted to reduce excess mortality to 5% and 0% in low-to-middle-income countries and high-income countries, respectively, could save nearly ten million lives by 2050.

**What Do These Findings Mean?:**

In estimating the global burden of SCA in children under five years old between 2010 and 2050 and the number of newborn lives that could be saved by implementation of health interventions, the researchers made numerous assumptions reflected in the uncertainty associated with the projections. For example, they assumed that implementation of specific interventions would lead to an immediate reduction of excess mortality in newborns with SCA. The study's findings confirm, however, that the global burden of SCA is increasing and indicate that the implementation of specific interventions could extend the lives of millions of newborns with SCA. Although further studies are needed to assess the current and future health care needs of children with SCA, these findings highlight the need to develop and implement national public health planning and funding policies for SCA, particularly in low- and middle-income countries.

**Additional Information:**

Please access these websites via the online version of this summary at http://dx.doi.org/10.1371/journal.pmed.1001484.

- This study is further discussed in a *PLOS Medicine* Perspective by Edward Fottrell and David Osrin
- The US National Heart, Lung, and Blood Institute provides detailed information (including personal stories) about sickle cell anemia (in English and Spanish)
- The UK National Health Service Choices website also provides detailed information and a personal story about sickle cell anemia
- The Sickle Cell Society, a UK-based not-for-profit organization, provides information for patients and carers and includes a children's website
- The World Health Organization has a factsheet on sickle cell anemia and other inherited hemoglobin diseases (in several languages)
- MedlinePlus provides links to further resources about sickle cell anemia (in English and Spanish)
- The Malaria Atlas Project provides epidemiological information on the inherited blood disorders (including sickle cell anemia) that affect our response to malaria infection
- The Global Sickle Cell Disease Network is a portal bringing together leading sickle cell disease researchers and clinicians from high-, middle-, and low-income countries to form a network

## Introduction

While considerable efforts are currently being invested into reducing the global burden of infectious diseases, particularly malaria, tuberculosis, and HIV [1],[2], the burden of birth defects has largely been neglected [3]–[5]. It has recently been estimated that more than 7 million babies are born each year with either a congenital abnormality or a genetic disease [3]. Five disorders constitute approximately 25% of these births, two of which, haemoglobinopathy and glucose-6-phosphate dehydrogenase deficiency, are monogenic diseases [6].

Amongst the haemoglobinopathies, sickle cell disease is by far the largest public health concern. Sickle haemoglobin (HbS) is a structural variant of normal adult haemoglobin (HbA) that is inherited as an autosomal recessive Mendelian trait. While heterozygote individuals are generally asymptomatic, homozygote individuals (i.e., those with SCA) suffer from lifelong acute and chronic complications [7]. Although sickle cell disorders include not only SCA but also co-inherited haemoglobin S and haemoglobin C (HbSC disease) or β-thalassaemia (HbS/β-thalassaemia), the present study focuses exclusively on SCA, the most severe and most common globally, accounting for an estimated 83% of all newborns with sickle cell disorders [8].

Because of evolutionary selection due to malaria protection, the highest frequencies of SCA are seen in tropical regions [9]. The vast majority of newborns with SCA occur in low- and middle-income countries. Without early diagnosis and treatment, most of those affected die during the first few years of life, with reported excess mortality reaching up to 92% [10]. Furthermore, infectious diseases have a role in causing increased severity of SCA [11],[12]. As low- and middle-income countries go through epidemiological transition and improve hygiene, nutrition, and public health policies and infrastructures, impressive reductions in overall infant and childhood mortality have started to be observed [13]–[16].

Following population migration, SCA is now seen throughout the world, as illustrated by the implementation of universal screening programmes in the United States of America, in the United Kingdom, and in French overseas territories. As it seems likely that human migration will continue to increase with further globalisation [17], the implementation of prevention measures, including diagnosis and counselling, in low- and middle-income countries will be of direct relevance for high-income countries.

Awareness about the clinical and economic burden of SCA is rising, albeit slowly. In 2006, the World Health Organization (WHO) recognised SCA as a global public health problem [18]. In 2010, the 63rd World Health Assembly adopted a resolution on the prevention and management of birth defects, including sickle cell disease and the thalassaemias. Finally, haemoglobinopathies have been included in the most recent Global Burden of Diseases, Injuries, and Risk Factors Study (the GBD 2010 study), which aims at providing a comprehensive and systematic evidence-based assessment of the burden of major diseases and injuries [19].

Quantitative studies provide essential evidence on which to base public health decisions [20]. No such studies are currently available for either SCA or other birth defects. We recently estimated national allele frequencies for HbS using a contemporary database of representative population surveys and a Bayesian geostatistical framework [21]. By combining our estimates with high-resolution data on crude birth rates and population densities, we were able to estimate the global number of SCA-affected births by country for 2010. Here, we use these estimates and demographic projections to (i) assess the magnitude of the expected increase in the global burden of SCA between 2010 and 2050, (ii) identify the countries most likely to be affected by changes over the next decades, and (iii) provide quantitative evidence to guide public health decisions at global, regional, and national scales.

## Methods

**Figure 1:**
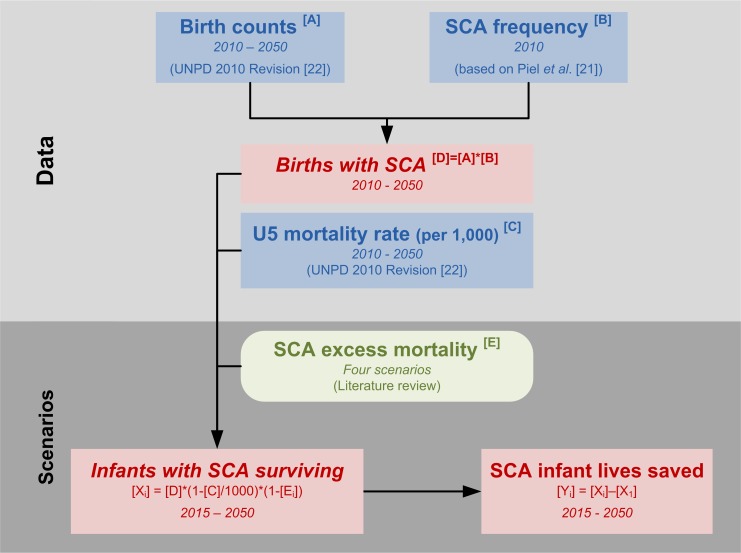
Schematic overview of our model approach. Definition of variables: *A*, birth counts; *B*, frequency of SCA; *C*, mortality rate in under-five children; *D*, number of births with SCA; *E*, excess mortality in under-five children with SCA; *i*, scenario number, from 1 to 4; *X*, number of infants with SCA surviving; *Y*, number of lives of infants with SCA saved. U5, under five; UNPD, United Nations Population Division World Population Prospects [22].

We conducted a quantitative investigation of the trends in the number of newborns with SCA at national, regional, and global scales. We then used a scenario-based approach that accounted for differences between low-, middle-, and high-income countries. Population movements are not considered in this study because of their unpredictable nature and a lack of systematic data for their prediction at the global level. Our model approach is summarised in Figure 1, and a worked example showing how values were calculated for Nigeria is presented in Table S1. A summary of the assumptions made in this study is shown in Table 1.

**Table 1:** Summary of the assumptions and limitations of this study.

Assumption	Notes/Limitations
We assumed that allele frequencies were constant over the study period (2010–2050).	This is based on the slow kinetics of inherited disorders and on data from Jamaica [25], but it neglects the influence of population migrations because of their unpredictable nature.
We have assumed the implementation of specific health interventions in 2015 to calculate the number of lives that could be saved.	Although some countries are currently considering implementing specific interventions for SCA, it is impossible to predict when each country might implement such interventions and to which extent.
We assumed that overall trends in the burden of SCA were driven by newborns.	Data on the prevalence of SCA in adults is very limited, both in high- and low-income countries. Moreover, few studies have investigated SCA survival in adults.
We assumed that it is possible to reduce excess mortality in under-five children to zero in high-income countries and to 5% in low- and middle-income countries.	This is based on data from large-scale studies conducted in the United States, the United Kingdom, and Jamaica, summarised in Quinn et al. [34].
We assumed that information on consanguinity was too crude to be incorporated.	There is currently no global and comprehensive database on consanguinity.
We assumed that the implementation of specific interventions would lead to an immediate reduction of the excess mortality in newborns with SCA.	This is supported by the proven efficacy of these interventions. Nevertheless, the rapidity with which they might be implemented may vary widely between countries.
We assumed that focused care for children under 5 y would not detract from care for others, including parents and older patients with SCA.	In the short term, improving the health of children under 5 y with SCA would increase awareness about this disease, which would inevitably benefit adults and older patients with SCA. In the long term, early diagnosis and appropriate health care helps prevent many of the serious clinical complications observed in adults with SCA.
We assumed that data on the costs of implementing specific health interventions were too limited, particularly in low- and middle-income countries, to be incorporated into this study.	Although data on the costs of these health interventions are available from various high-income countries, we could not find any published study presenting such data for low- and middle-income countries.

### Projected Number of Newborns with SCA

Our projections of the number of newborns with SCA are based on the product of estimates of SCA frequency and projected birth counts. For SCA, we have used the median and interquartile range—the interval between the 25% and 75% quantiles of the predicted posterior distribution—of our own frequency estimates for 2010 [21]. Although only estimates of allele frequencies were previously published, SCA frequencies were also calculated within the Bayesian geostatistical framework used. For birth counts, we used medium-, low-, and high-fertility variant projections for 5-y periods between 2010 and 2050 from the 2010 revision of the United Nations World Population Prospects [22]. The lower bound of our confidence intervals (CIs) is based on the 25% quantile for SCA estimates and the low-fertility variant for birth counts. The higher bound of our CIs is based on the 75% quantile for SCA estimates and the high-fertility variant for birth counts. Data, with CIs, are presented for each country and for WHO regions, HbS regions (as defined in [21]), and the world in Table S2.

**Figure 2:**
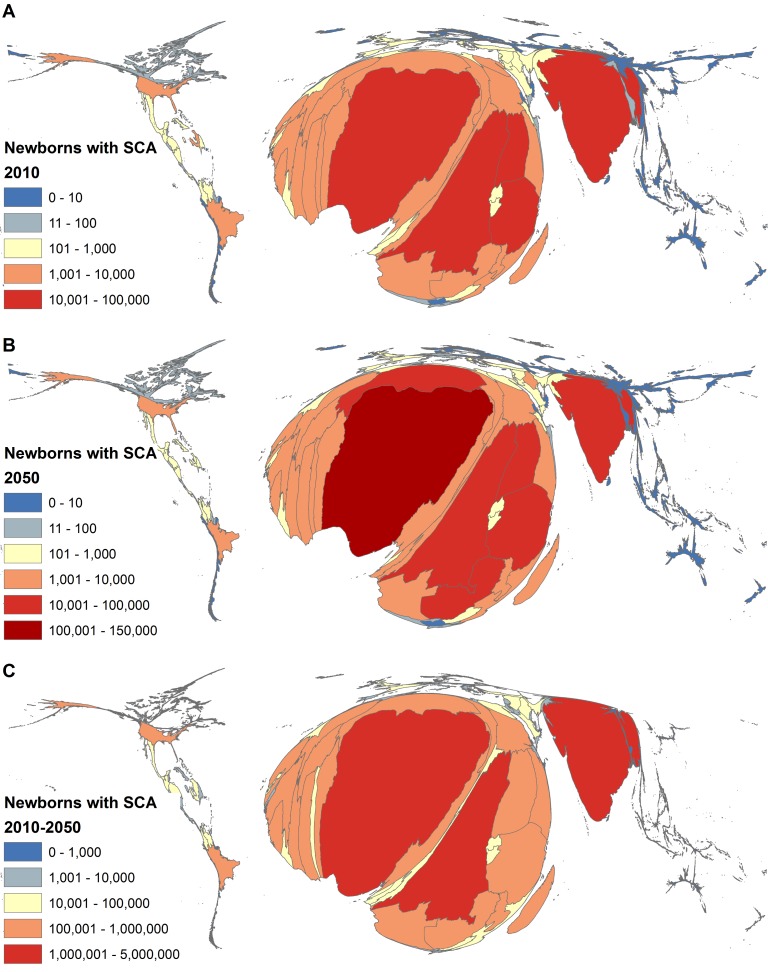
Cartograms of the estimated number of newborns with SCA per country. Cartograms of the estimated number of newborns with SCA per country in 2010 (A), 2050 (B), and overall from 2010 to 2050 (C), based on data presented in Table S2. The estimates are based on the median of the posterior predictive distribution for SCA frequencies generated by our Bayesian geostatistical model described in Piel et al. [21] and the medium-fertility variant of the birth projections from the 2010 revision of the UN World Population Prospects [22].

We generated cartograms of the number of newborns with SCA in 2010, 2050, and over the period studied (2010–2050) (Figure 2) using the Cartogram Geoprocessing Tool in ArcGIS 10.1 (Esri). Cartograms are maps distorted proportionally to a variable other than land area or geographical space [23],[24]. They help to draw attention to regions or countries that are overrepresented or underrepresented when considering the particular variable mapped.

**Figure 3:**
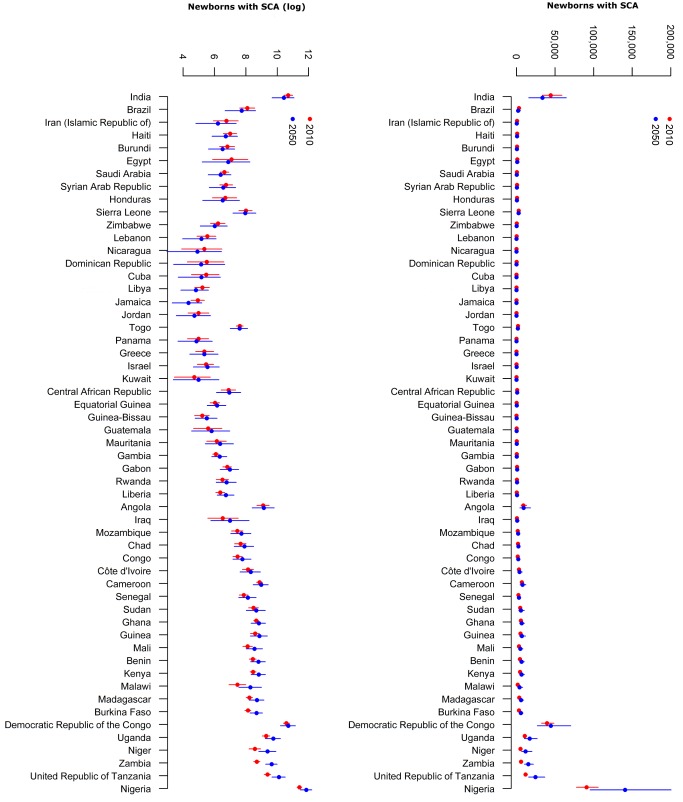
Country ranking based on estimated number of newborns with SCA in 2010 and 2050. Limited to countries for which the estimated median SCA frequency in 2010 was higher than 0.001 and the estimated number of newborns with SCA in 2010 was higher than 100.

We then ranked countries based on the magnitude of the absolute change in the estimated median number of newborns with SCA born between 2010 and 2050 (Table S2). Countries in which the increase in the number of newborns with SCA was the highest over the study period were assigned the lowest rank, while countries in which the decrease in the number of newborns with SCA was the highest over the study period were assigned the highest rank. Ranks are shown in Table S2. For illustrative purposes, we have limited this analysis to countries with a SCA frequency higher than 0.001 and in which more than 100 newborns with SCA were estimated for 2010 (Figure 3A). We applied a logarithmic transformation to further illustrate relative changes (Figure 3B).

### Lives Saved Scenarios

Because of the inheritance mechanism of the sickle cell gene, changes in SCA allele frequency occur slowly, over generations [23],[24]. Studies conducted in Jamaica suggest that even in the absence of positive selection for heterozygotes the prevalence of newborns with SCA will remain stable over very long periods of time [25]. For the purposes of this analysis, we therefore assumed that the prevalence of newborns with SCA will remain constant during the period under study (2010–2050). Although few data are available regarding SCA mortality, particularly in the areas of highest prevalence, sharp reductions in SCA mortality in young children following the implementation of specific health measures are well documented in the US [26],[27] and Jamaica [28],[29]. Having calculated the baseline number of expected newborns with SCA at global, regional, and national scales by 5-y intervals from 2010–2050, we then tested the following four scenarios assuming the implementation in 2015 of the health measures described. (i) Scenario 1 represents our best assessment of the current situation: that in low- and middle-income countries, where the public health infrastructures required for the diagnosis and care of children with SCA are weak or absent, there is a 90% excess mortality among children under five with SCA, based on data from Fleming et al. [30] and Grosse et al. [10], but that in high-income countries with good access to public health infrastructures, the excess mortality is only 10%, based on data from Platt et al. [31]. Excess mortality is calculated as the difference between the frequency of SCA in newborns and in 5-y-olds, divided by the frequency of SCA in newborns [10]. The number of surviving children with SCA by age 5 y is therefore calculated as the number of newborns with SCA multiplied by the survival rate in the overall under-five population multiplied by the complement of the excess mortality in children with SCA (1−*m*_excess_). (ii) Scenario 2 represents a realistic short-term aim: to reduce the excess mortality to 50% in low- and middle-income countries, as described in Simpore et al. [32] and Grosse et al. [10] and to 5% in high-income countries, reflecting basic improvements in general public health infrastructures in both sets of countries. It seems likely that such improvements could be achieved by making penicillin prophylaxis and screening programmes or prenatal diagnosis widely available, [29]. (iii) Scenario 3 represents an optimistic aim that could correspond to the implementation of specific health measures targeting patients with SCA, such as widespread screening and the provision of specialised clinics: to reduce excess mortality to 10% in low- and middle-income countries and to eliminate it in high-income countries, based on recent data from Quinn et al. and Telfer et al. [33]–[35]. (iv) Scenario 4 represents the situation where a 5% excess mortality is observed in low- and middle-income countries and no excess mortality is observed in high-income countries [33]–[35]. A summary of these scenarios is presented in Table 2. By comparing Scenarios 2, 3, and 4 to Scenario 1, we calculated the number of lives that could be saved for the different levels of interventions considered.

**Table 2:** Summary of the level of public health infrastructure and excess mortality considered per income class and for each of the four scenarios tested.

Scenario	Low-/Middle-Income Countries (GNI_pc_≤US$12,275)		High-Income Countries (GNI_pc_>US$12,275)	
	General Level of Public Health Infrastructures for Under-Five Children with SCA	Excess Mortality in Under-Five Children with SCA	General Level of Public Health Infrastructures for Under-Five Children with SCA	Excess Mortality in Under-Five Children with SCA
Scenario 1	Poor access to public health infrastructures	90%	Good access to public health infrastructures	10%
Scenario 2	Good access to public health infrastructures	50%	Specific interventions for children with SCA (e.g., diagnosis, treatment)	5%
Scenario 3	Specific interventions for children with SCA (e.g., diagnosis, treatment)	10%	Universal screening programme (optimum)	0%
Scenario 4	Universal screening programme	5%	Universal screening programme (optimum)	0%

Although the World Population Prospects [22] include migration data, only predictions of the net number of migrants are presented. Such data do not allow quantifying future fluxes between countries, which would be required for inclusion in the present study.

We classified countries in four categories: low, middle low, middle high, and high income, based on their 2010 gross national income per capita (GNI_pc_), converted into US dollars, as calculated by the World Bank (http://data.worldbank.org/indicator/NY.GNP.PCAP.CD), and the World Bank income group classes (low income, US$1,005 or less; lower middle income, US$1,006–US$3,975; upper middle income, US$3,976–US$12,275; and high income, US$12,276 or more). For our mortality baseline, we used the U5m medium-, low-, and high-fertility variant projections for 5-y periods between 2010 and 2050 from the 2010 revision of the UN World Population Prospects [22]. Our economic indicator was the projected gross domestic product per capita (GDP_pc_) as published in the French Research Center in International Economics's BASELINE database (http://www.cepii.fr/anglaisgraph/bdd/baseline.htm). Full data on U5m and GDP_pc_ are presented in Table S3.

**Figure 4:**
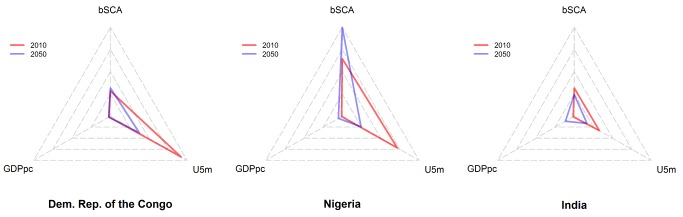
Radar plots of newborns with SCA, gross domestic product, and under-five mortality for the DRC, Nigeria, and India. Radar plots for the DRC (A), Nigeria (B), and India (C). bSCA, estimated number of newborns with SCA.

The capacity of countries to manage a changing number of newborns with SCA will depend on their current and future economic status and on the overall survival of children. To illustrate these changes, we created radar plots for each country displaying (i) the number of newborns with SCA based on United Nations medium-fertility variant projections, (ii) GDPpc, and (iii) U5m in 2010 and in 2050 (Figure 4). Radar plots represent an easy visualisation tool over time (within each country) and space (between countries). Moreover, they provide an appropriate, intuitive, and visually explicit ranking method for meta-analyses [36]. Each axis of the radar plots was scaled independently based on the minimum and maximum values of each indicator across all countries.

## Results

### Projected Births and Newborns with SCA

The world population is expected to increase from 6,896 million individuals in 2010 to 9,306 million in 2050 [22]. In many African countries, where SCA frequency is the highest [21], the overall number of births is expected to double during the period of time considered in this study [22]. As a consequence, when assuming constant gene frequencies, it is expected that the annual number of newborns with SCA, estimated to be 305,800 (CI: 238,400–398,800) globally in 2010, will likely increase by about one-third by 2050 (404,200 [CI: 242,500 (+2%)–657,600 (+65%)]) (Table 3). Globally, we estimated the overall number of births affected by SCA between 2010 and 2050 to be 14,242,000 (CI: 9,923,600–20,498,500).

**Table 3:** Projected number of newborns with SCA born in 2010 and 2050 for the three most affected countries (Nigeria, India, and the DRC), WHO regions, HbS regions, and worldwide.

Category	Sub-Category	2010			2050					2010–2050		
		Number of Newborns with SCA^a^	CI^b^	Percent of Category	Number of Newborns with SCA^c^ (Change from 2010)		CI^b^	Percent of Category (Change from 2010)		Number of Newborns with SCA^d^	CI^b^	Percent of Category
**Country**	Nigeria	91,011	[77,881–106,106]	29.8%	140,837	(+54.7)	[95,487–200,604]	34.8%	(+17.1)	4,600,639	[3,566,180–5,863,269]	32.3%
	India	44,425	[33,692–59,143]	14.5%	33,890	(−23.7)	[15,936–64,740]	8.4%	(−42.3)	1,605,013	[1,007,436–2,493,101]	11.3%
	DRC	39,743	[32,593–48,788]	13.0%	44,663	(+12.4)	[27,062–70,542]	11.1%	(−15.0)	1,761,226	[1,281,666–2,405,181]	12.4%
**WHO region** e	AFRO	237,381	[191,067–295,354]	77.6%	347,674	(+46.5)	[217,838–536,072]	86.0%	(+10.8)	11,697,397	[8,461,417–16,020,136]	82.1%
	AMRO	11,143	[6,305–19,823]	3.6%	9,596	(−13.9)	[3,503–23,899]	2.4%	(−34.9)	417,065	[195,281–862,232]	2.9%
	EMRO	10,559	[6,242–19,390]	3.5%	10,791	(+2.2)	[4,529–26,235]	2.7%	(−22.7)	433,457	[223,215–897,309]	3.0%
	EURO	1,939	[932–4,330]	0.6%	1,902	(−1.9)	[604–5,717]	0.5%	(−25.8)	75,897	[30,533–192,299]	0.5%
	SEARO	44,454	[33,696–59,338]	14.5%	33,910	(−23.7)	[15,938–64,943]	8.4%	(−42.3)	1,605,997	[1,007,529–2,501,090]	11.3%
	WPRO	6	[1]–[23]	0.0%	7	(+16.7)	[1]–[36]	0.0%	(−11.7)	249	[46–1,122]	0.0%
**HbS region** f	Eurasia	5,130	[2,474–11,179]	1.7%	4,478	(−12.7)	[1,385–13,518]	1.1%	(−34.0)	193,796	[77,985–484,394]	1.4%
	Americas	11,181	[6,324–19,896]	3.7%	9,628	(−13.9)	[3,514–23,983]	2.4%	(−34.9)	418,472	[195,879–865,325]	2.9%
	Sub-Saharan Africa	242,187	[194,549–302,012]	79.2%	353,533	(+46.0)	[220,901–546,741]	87.5%	(+10.4)	11,916,113	[8,599,975–16,361,830]	83.7%
	Southeast Asia	7	[1]–[32]	0.0%	8	(+14.3)	[1]–[46]	0.0%	(−13.5)	279	[48–1,503]	0.0%
	Arab-India	47,264	[35,050–65,640]	15.5%	36,540	(−22.7)	[16,730–73,326]	9.0%	(−41.5)	1,713,342	[1,049,712–2,784,723]	12.0%
**World**		305,773	[238,400–398,775]	100%	404,190	(+32.2)	[242,530–657,634]	100%	—	14,242,002	[9,923,623–20,498,521]	100%

Complete data for all countries are presented in Table S1. Proportions per category are indicated for the predicted newborns with SCA. Relative changes are shown within parentheses.

a Calculated as the product between the median SCA frequency based on the model outputs described in Piel et al. [21] and the births per year for 2010–2015 from the 2010 revision of the UN World Population Prospects [22].

b CIs based on the interquartile range of the SCA frequency estimates and the low- and high-fertility variants for birth counts.

c Calculated as the product between the median SCA frequency based on the model outputs described in Piel et al. [21], assuming constant allele frequencies over the study period and using the data on births per year for 2050–2055 from the 2010 revision of the UN World Population Prospects [22].

d Total estimated newborns with SCA born between 2010 and 2050.

e As defined at http://www.who.int/about/regions/en/index.html. AFRO, African Region; AMRO, Region of the Americas; EMRO, Eastern Mediterranean Region; EURO, European Region; SEARO, Southeast Asia Region; WPRO, Western Pacific Region.

f As shown in Web Figure 8 of Web Appendix 2 of Piel et al. [21].

Regionally, in 2010, an estimated 79% (242,200 [CI: 194,500 (82%)–302,000 (76%)]) of newborns with SCA occurred in sub-Saharan Africa (Table 3; Figure 2A). This proportion is expected to increase to 88% (353,500 [CI: 220,900 (91%)–546,700 (83%)]) by 2050 (Table 3; Figure 2B). In contrast, based on the UN demographic projections, the proportion of newborns with SCA in the other HbS regions (Eurasia, the Americas, and Arab-India), apart from Southeast Asia, where SCA burden is very small, is expected to decrease (Table 3).

**Figure 5:**
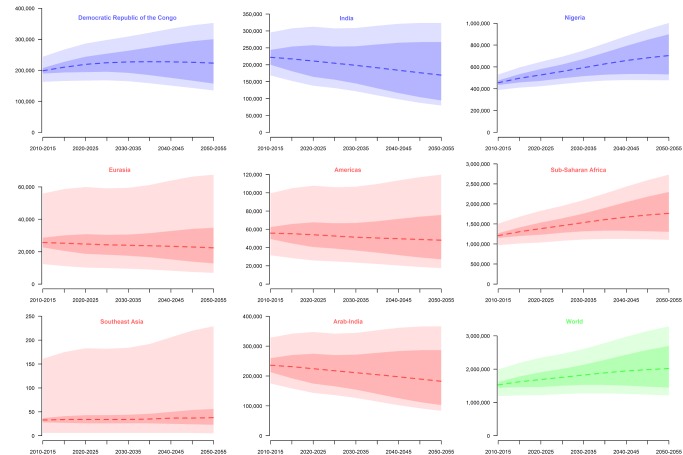
Projections of estimated newborns with SCA between 2010 and 2050. Projections of estimated number of newborns with SCA (*y*-axis) between 2010 and 2050 for the DRC, India, and Nigeria (in blue); HbS regions: Eurasia, the Americas, sub-Saharan Africa, Southeast Asia, and Arab-India (in red; defined in Piel et al. [21]); and globally (in green). The dark-shaded areas represent the uncertainty in the demographic data. The light-shaded areas show the uncertainty associated with our estimates of SCA frequency.

In 2010, we estimated that three countries (Nigeria, India, and the Democratic Republic of the Congo [DRC]) represented 57% (175,200 [CI: 144,200 (60%)–214,000 (54%)]) of the annual number of newborns with SCA globally (305,800 [CI: 238,400–398,800]). By 2050, these countries are projected to represent 55% (219,400 [CI: 138,500–335,900] amongst 404,200 [CI: 242,500–657,600]). But while the relative contribution of Nigeria is projected to increase from 30% (91,000 [CI: 77,900 (33%)–106,100 (27%)]) to 35% (140,800 [CI: 95,500 (39%)–200,600 (31%)]), the DRC's and India's relative contributions are expected to decrease from 13% (39,700 [CI: 32,600 (14%)–48,800 (12%)]) to 11% (44,700 [CI: 27,100 (11%)–70,500 (11%)]) and from 15% (44,400 [CI: 33,700 (15%)–59,100 (12%)]) to 8% (33,900 [CI: 15,900 (7%)–64,700 (10%)]), respectively. Projections for the three most affected countries, regions, and the world are plotted in Figure 5. Estimates for 2010, 2050, and 2010–2050 at national, regional, and global scales are presented in Table S2 and plotted for selected countries in Figure 3.

### Estimated SCA Deaths and Lives Saved

By comparing the results of our scenarios with additional interventions (Scenarios 2, 3, and 4) with the scenario based on current practice (Scenario 1), we estimated the number of lives that could be saved at different scales (Tables 4 and S1). A global transition from Scenario 1 to Scenario 2 in 2015, would save 113,500 [CI: 85,100–152,900] newborns in 2015 and a total of 5,302,900 [CI: 3,174,800–6,699,100] newborns by 2050. A similar transition to Scenario 3 would almost double the number of newborns saved in 2015 and overall between 2015 and 2050. Transitioning to Scenario 4 in 2015 would save 241,100 [CI: 180,800–324,900] newborns with SCA in 2015 and a total of 9,806,000 [CI: 6,745,800–14,232,700] by 2050.

**Table 4:** Estimated number of lives saved of children with SCA in 2015, in 2050, and over the period 2015–2050 when comparing scenarios with reduced excess mortality (Scenarios 2, 3, and 4) to a status quo scenario (Scenario 1), based on the implementation of measures in 2015.

Category	Sub-category	Scenario 2 versus Scenario 1^a^			Scenario 3 versus Scenario 1^b^			Scenario 4 versus Scenario 1^c^		
		Lives Saved in 2015 [CI]	Lives Saved in 2050 [CI]	Total Lives Saved (2015–2050) [CI]	Lives Saved in 2015 [CI]	Lives Saved in 2050 [CI]	Total Lives Saved (2015–2050) [CI]	Lives Saved in 2015 [CI]	Lives Saved in 2050 [CI]	Total Lives Saved (2015–2050) [CI]
**Country**	Nigeria	34,741[28,773–41,690]	53,549[36,306–76,274]	1,711,430[1,160,127–1,952,218]	69,482[57,547–83,380]	397,648[336,247–76,274]	3,031,798[2,320,254–3,904,437]	73,825[61,144–88,591]	113,792[77,151–162,083]	3,221,285[2,465,270–4,148,464]
	India	16,361[11,454–23,155]	13,092[6,157–25,010]	630,710[319,393–837,820]	32,721[22,908–46,309]	120,817[86,408–25,010]	1,053,817[638,786–1,675,640]	34,766[24,340–49,204]	27,821[13,083–53,147]	1,119,681[678,710–1,780,368]
	DRC	14,018[11,067–17,793]	16,519[10,009–26,091]	627,816[390,316–757,135]	28,036[22,135–35,587]	155,491[123,659–26,091]	1,092,724[780,632–1,514,270]	29,788[23,518–37,811]	35,104[21,270–55,444]	1,161,019[829,421–1,608,911]
**WHO region** d	AFRO	89,925[69,859–115,437]	132,115[82,801–203,647]	4,355,115[2,740,435–5,321,803]	179,850[139,719–230,874]	1,002,895[793,528–203,647]	7,684,321[5,480,870–10,643,607]	191,088[148,449–245,300]	280,741[175,951–432,746]	8,164,498[5,823,366–11,308,690]
	AMRO	3,251[1,618–6,398]	2,587[857–6,922]	136,864[44,075–228,974]	6,502[3,236–12,796]	16,193[9,857–6,922]	206,191[88,149–457,948]	6,890[3,428–13,564]	5,475[1,812–14,663]	218,379[93,295–485,295]
	EMRO	3,692[2,034–7,249]	3,896[1,642–9,524]	161,779[67,300–285,853]	7,384[4,068–14,499]	28,836[17,542–9,524]	268,526[134,600–571,706]	7,838[4,318–15,393]	8,273[3,487–20,225]	285,083[142,896–607,008]
	EURO	225[110–528]	194[65–594]	15,558[3,140–18,794]	449[220–1,056]	−1,360[307–594]	14,596[6,280–37,588]	468[230–1,099]	402[136–1,232]	15,168[6,545–39,061]
	SEARO	16,371[11,455–23,233]	13,100[6,157–25,090]	631,107[319,422–840,559]	32,743[22,910–46,466]	120,887[86,415–25,090]	1,054,474[638,845–1,681,117]	34,789[24,342–49,371]	27,838[13,084–53,317]	1,120,379[678,773–1,786,187]
	WPRO	0[0–2]	0[0–2]	38[2]–[61]	1[0–3]	−9[−1–2]	22[4–122]	1[0–3]	1[0–4]	22[4–124]
**HbS region** e	Eurasia	1,322[587–3,108]	1,081[322–3,396]	59,763[16,530–111,629]	2,643[1,173–6,217]	5,432[3,544–3,396]	85,488[33,061–223,257]	2,796[1,241–6,578]	2,285[681–7,181]	90,406[34,959–236,177]
	Americas	3,266[1,625–6,428]	2,599[861–6,956]	137,441[44,274–230,058]	6,531[3,250–12,856]	16,294[9,907–6,956]	207,157[88,548–460,115]	6,921[3,442–13,628]	5,502[1,821–14,735]	219,406[93,719–487,597]
	Sub-Saharan Africa	91,802[71,156–118,143]	134,390[83,991–207,790]	4,439,957[2,786,376–5,438,922]	183,603[142,312–236,286]	1,020,601[805,975–207,790]	7,832,117[5,572,752–10,877,845]	195,076[151,205–251,051]	285,577[178,479–441,549]	8,321,531[5,920,991–11,557,568]
	Southeast Asia	1[0–5]	1[0–6]	50[2–192]	1[0–11]	−7[0–6]	42[5–383]	1[0–11]	1[0–13]	44[5–401]
	Arab-India	17,107[11,723–25,232]	13,852[6,358–27,723]	665,630[327,632–918,006]	34,213[23,446–50,464]	124,906[88,233–27,723]	1,105,597[655,264–1,836,012]	36,345[24,908–53,608]	29,431[13,509–58,900]	1,174,501[696,117–1,950,391]
**GNI_pc_** f	Low	41,282[30,901–54,893]	59,152[35,094–95,569]	1,985,336[1,194,615–2,523,028]	82,564[61,802–109,785]	459,730[348,359–95,569]	3,500,023[2,389,229–5,046,056]	87,724[65,664–116,647]	125,699[74,575–203,084]	3,718,774[2,538,556–5,361,435]
	Middle low	68,632[52,343–91,080]	89,846[55,395–142,739]	3,153,383[1,929,209–3,929,860]	137,265[104,687–182,161]	694,920[548,682–142,739]	5,503,387[3,858,418–7,859,720]	145,844[111,230–193,546]	190,922[117,715–303,320]	5,847,349[4,099,569–8,350,952]
	Middle high	3,224[1,663–6,230]	2,544[898–6,586]	121,528[45,082–217,854]	6,448[3,326–12,461]	21,328[11,517–6,586]	201,353[90,164–435,708]	6,851[3,534–13,240]	5,405[1,909–13,995]	213,938[95,799–462,940]
	High	315[171–582]	336[136–780]	40,794[5,538–22,640]	630[343–1,164]	−9,083[−993–780]	22,607[11,076–45,280]	630[343–1,164]	673[272–1,560]	22,607[11,076–45,280]
**World**		113,498[85,091–152,924]	151,925[91,533–245,879]	5,302,904[3,174,823–6,699,064]	226,996[170,183–305,849]	1,167,238[907,660–245,879]	9,230,508[6,349,646–13,398,128]	241,143[180,798–324,891]	322,799[194,490–522,395]	9,806,002[6,745,807–14,232,681]

a Calculated as the difference between the number of newborns with SCA surviving in Scenario 2 (50% and 5% excess mortality in low- and middle-income countries and high-income countries, respectively) and in Scenario 1 (90% and 10% excess mortality in low- and middle-income countries and high-income countries, respectively). CIs are based on the interquartile range of the SCA estimates and the low- and high-fertility variants of the projected birth counts.

b Calculated as the difference between the number of newborns with SCA surviving in Scenario 3 (10% and 0% excess mortality in low- and middle-income countries and high-income countries, respectively) and in Scenario 1 (90% and 10% excess mortality in low- and middle-income countries and high-income countries, respectively). CIs are based on the interquartile range of the SCA estimates and the low- and high-fertility variants of the projected birth counts.

c Calculated as the difference between the number of newborns with SCA surviving in Scenario 4 (5% and 0% excess mortality in low- and middle-income countries and high-income countries, respectively) and in Scenario 1 (90% and 10% excess mortality in low- and middle-income countries and high-income countries, respectively). CIs are based on the interquartile range of the SCA estimates and the low- and high-fertility variants of the projected birth counts.

d As defined at http://www.who.int/about/regions/en/index.html. AFRO, African Region; AMRO, Region of the Americas; EMRO, Eastern Mediterranean Region; EURO, European Region; SEARO, Southeast Asia Region; WPRO, Western Pacific Region.

e As shown in Web Figure 8 of Web Appendix 2 of Piel et al. [21].

f GNI_pc_ in US dollars, based on the World Bank classification: low, US$1,005 or less; middle low, US$1,006–US$3,975; middle high, US$3,976–US$12,275; and high, US$12,276 or more.

The vast majority of SCA-affected newborn lives that could be saved occur in sub-Saharan Africa. This is obvious when looking at the annual estimates for 2010 and 2050, but even more striking when looking at the overall calculations of lives lost over the 35-y projected period from 2015 to 2050 (Table 4). Nigeria's contribution to the global burden of SCA is particularly important. Based on GNI_pc_, more than 95% of the mortality burden among newborns with SCA will fall to low- and middle-low-income countries (Table 4).

Our radar charts illustrate the different types of challenges that are faced by different countries (Figures 4 and S1). For example, in the DRC, the expected increase in the number of newborns with SCA (from 39,800 [CI: 32,600–48,800] to 44,700 [CI: 27,100–70,500]) will probably be accompanied by significant improvements in survival (reduction of U5m from 180 to 75 per 1,000). This will result in growing pressure on health care services during a period in which the economic status of the country is not expected to experience significant improvements (an increase in the GDP_pc_ from US$3,812 to only US$4,135) (Figure 4A; Table S3). Nigeria will likely see a very large increase in the number of newborns with SCA (from 91,000 [CI: 77,900–106,100] in 2010 to 140,800 [CI: 95,500–200,600] in 2050), while U5m is projected to decrease from 141 to 49 per 1,000. This will aggravate the national health burden of SCA to an extent that will be poorly compensated by the projected increase in its GDP_pc_ from US$2,137 to US$9,015 (Figure 4B; Table S3). Conversely, the situation seems a little less alarming in India, where we project that the number of newborns with SCA will decrease from 44,400 (CI: 33,700–59,100) to 33,900 (CI: 15,900–64,700), while GDP_pc_ will increase by 6-fold (from US$3,062 to US$19,553) (Figure 4C; Table S3), a situation that will potentially make the SCA burden more manageable in that country.

## Discussion

Despite slowly growing awareness about haemoglobinopathies, and sickle cell disorders in particular, epidemiological data on the prevalence and burden of these disorders are still lacking [6],[21],[37]. In difficult economic times, evidence-based studies to support public health decisions and spending become increasingly important [38]. It has been suggested that the burden of haemoglobinopathies is going to increase over the coming decades [6],[39]. Such an increase is largely driven by two factors: population growth and public health transition. This study is, to our knowledge, the first attempt to quantify the magnitude of such increases based on existing epidemiological estimates and demographic projections. Our study highlights major inequalities in the current global distribution of newborns with SCA that are unlikely to be reduced in the coming decades. Basic interventions targeted to the most affected countries could save the lives of almost 10 million children born with SCA in the next 35 y.

The countries most affected face major challenges [37]. Currently, excess mortality in SCA patients in many low- and middle-income countries—most of whom remain undiagnosed—is extremely high, and SCA is often neglected in public health policies. It is anticipated that as overall U5m begins to fall because of improved nutrition and medical facilities, an increasing proportion of children under 5 y with SCA will survive long enough to reach medical attention. Public health improvements (including widespread use of prophylactic penicillin and vaccination) will help an increasing proportion of these children to survive through childhood and adulthood, and therefore to present for diagnosis and treatment (e.g., hydroxyurea, hospitalisation, transfusion) generating lifelong costs. The lack of interventions will inevitably lead to a large burden on the public health infrastructures and budgets of the countries most affected [40],[41]. This needs to be acknowledged by policy makers so that adequate planning can be used to save lives and keep treatment costs manageable. In the short term, the priority in countries with a high frequency of HbS is to identify affected births in order to provide appropriate treatment. Cheap diagnostic methods are available for HbS, and the cost-effectiveness of screening programmes has already been demonstrated [42],[43]. This study suggests that huge numbers of lives could be saved in Nigeria [44]–[46], the DRC [47]–[49], and India [50]–[52]. Some pilot screening programmes have recently started in these countries, but nationwide programmes are needed for a significant public health impact. In the long term, in the absence of a definitive treatment for SCA, the best intervention to reduce excess mortality caused by this disorder and to keep public health costs associated with the follow-up care of SCA patients through their lifetime manageable, especially in low-income countries, is to avoid the births of affected newborns [6],[37]. A good knowledge of individual status, including carriers, combined with a good education system about the inheritance mechanism of HbS and the risks associated might help reduce the number of newborns with SCA, but current evidence suggests that prenatal diagnosis and genetic counselling is more effective [40],[53]–[55]. Because of population diaspora, increasing admixture, and the absence of clinical symptoms in carriers, at-risk couples include a much larger subset of the population than originally estimated. Nevertheless, the idea that this disorder is confined to individuals of African origins is still common amongst the medical community, and this needs to change. Finally, it is essential to implement systems for monitoring temporal and spatial changes in the frequency of such disorders. In order to assess the efficiency of implemented policies, such systems need to collect reliable data from multiple health centres across a given country and to develop appropriate analytical methods.

Despite its novelty, this study has several limitations. First, it focuses only on newborns. Very few morbidity and mortality data are available for SCA patients, particularly in low-income countries, where deaths are usually attributed to other causes. Until universal screening at the population level is implemented, it is crucial to gather information on the mortality of SCA patients in order to define the public health and economic costs associated with HbS. It is likely that public health interventions such as those described here would result in indirect benefits for other age groups, but appropriate care for adults with SCA is something that also needs to be considered. Second, we have assumed that the implementation of specific interventions would lead to an immediate reduction of the excess mortality in newborns with SCA [27],[29],[56]. Although the benefits of these interventions have been clearly demonstrated in studies conducted in high-income countries, their implementation in low- and middle-income countries, in which general health infrastructures are poor, might be more challenging than assumed in this study. In addition, data on the costs of implementing interventions for children with SCA in low- and middle-income countries are currently lacking. Further studies on issues such as these will be needed before the optimal use of resources in different economic contexts can be defined. Third, the clinical phenotype of sickle cell disorders is very broad, being influenced by both genetic factors (e.g., α-thalassaemia or high levels of haemoglobin F) and environmental factors (e.g., infections) [57]. Moreover, there is some evidence to suggest that the Arab-Indian haplotype is milder than the African haplotypes [58]–[60]. If this is confirmed by large-scale population surveys, the economic burden in countries in which this haplotype is predominant might be lower than that in countries where the African haplotypes are prevalent. Current evidence was too sparse to account for this in the present study. Fourth, consanguineous marriages considerably increase the risk of having children with SCA in areas where the allele frequency of HbS is high. Such marriages are common in the Middle East and in various population groups worldwide, but only limited data are available globally [61],[62]. Finally, we assumed constant allele frequencies over the time period studied (40 y) [24],[25]. This assumption, which is based on the slow kinetics of inherited disorders, neglects the influence of population migrations. The magnitude and direction of past and current intra- or international population movements, often caused by political instability, civil disturbances, or environmental disasters, are difficult to assess. Allele frequencies within one country can be highly heterogeneous, making assumptions based only on nationality highly uncertain. Furthermore, it is almost impossible to predict future movements. Accessing immigration data [63] and using mobile phone network data [64],[65] for international migrations would be possible, but this is currently beyond the scope of this project.

### Conclusion

Multiple warnings regarding the effect of epidemiological and demographic transitions in low-income countries and their consequences for SCA burden have been published [6],[66]. By quantifying this increase from 2010 to 2050 using evidence-based data and identifying potential changes in the distribution of areas the most affected, we hope (i) to highlight further the need for greater awareness of SCA, appropriate public health policies, and funding; (ii) to guide the implementation of appropriate policies; and (iii) to provide a framework that could be applied to other birth defects. In most countries, the burden of SCA has so far not been recognised. Its long-term toll is nevertheless significant. These results highlight once more the need for further epidemiological collaborative studies, particularly in Nigeria, the DRC, and India, to define more accurately the current and future health burden of SCA.

## Supporting Information

Figure S1 **Radar plots for all countries.** bSCA, estimated number of newborns with SCA.
(PDF)

Table S1 **Worked example of our model approach for Nigeria (GNIpc: US$1,180; middle-low-income level).**
(PDF)

Table S2 **National, regional, and global SCA frequency (median and interquartile range), projected births (in thousands) with CI, and estimated number of newborns with SCA with CI.**
(PDF)

Table S3 **Indirect economic and mortality indicators per country.**
(PDF)

## Abbreviations

CI: confidence interval
DRC: Democratic Republic of the Congo
GDP_pc_: gross domestic product per capita
GNI_pc_: gross national income per capita
HbA: normal adult haemoglobin
HbS: sickle haemoglobin
SCA: sickle cell anaemia
U5m: under-five mortality
WHO: World Health Organization
