# Supplementary material for: Global Burden of Sickle Cell Anaemia in Children under Five, 2010–2050: Modelling Based on Demographics, Excess Mortality, and Interventions
Source: PLoS Med. 2013 Jul 16;10(7):e1001484. doi: 10.1371/journal.pmed.1001484 (PMC3712914; doi:10.1371/journal.pmed.1001484)
Supplement: Figure S1 — Radar plots for all countries. bSCA, estimated number of newborns with SCA. (PDF) [file pmed.1001484.s001.pdf]

Figure S1

Radar plots for all countries. bSCA, estimated number of newborns with SCA. GDPpc, gross domestic product per capita. U5m, under-five mortality

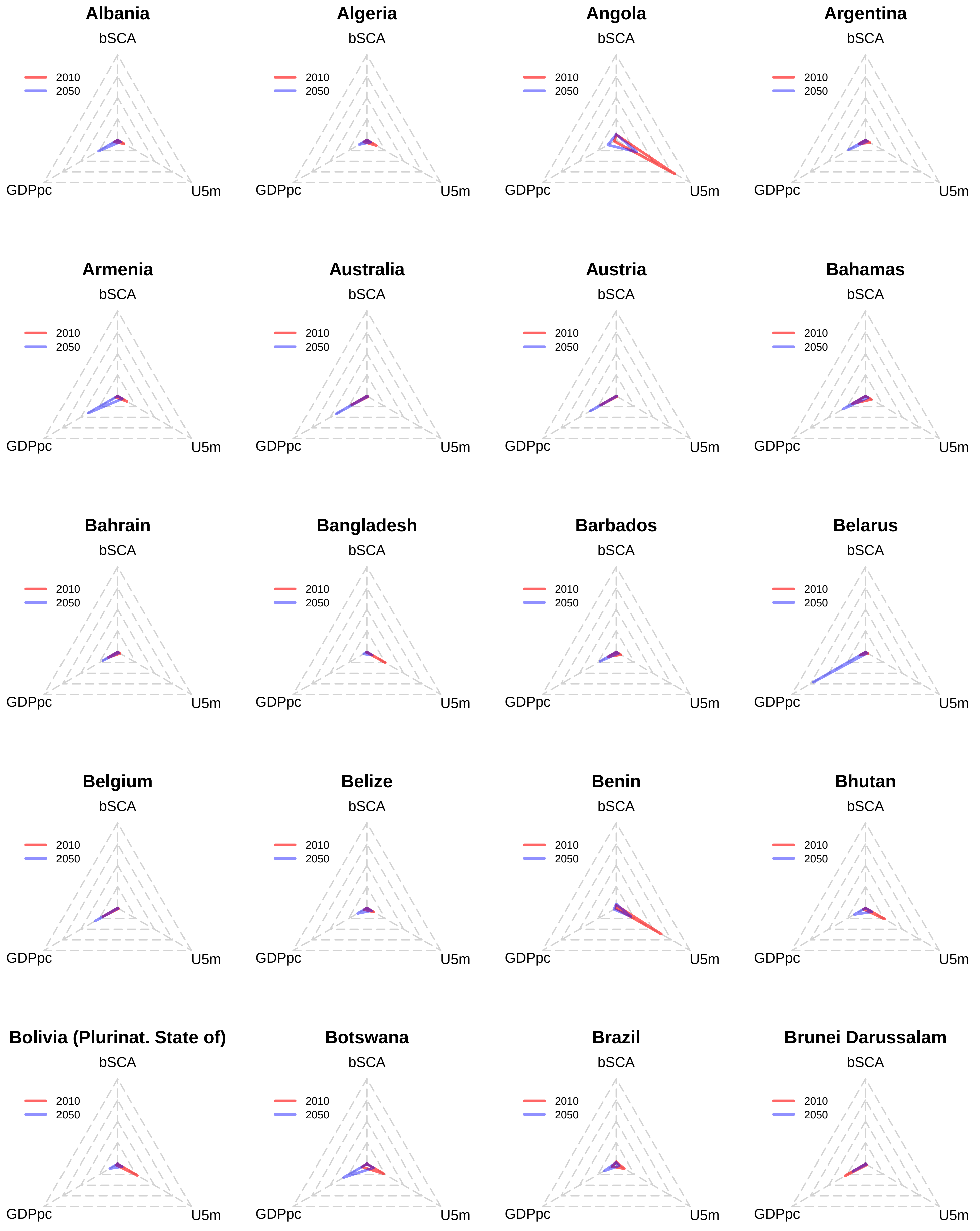

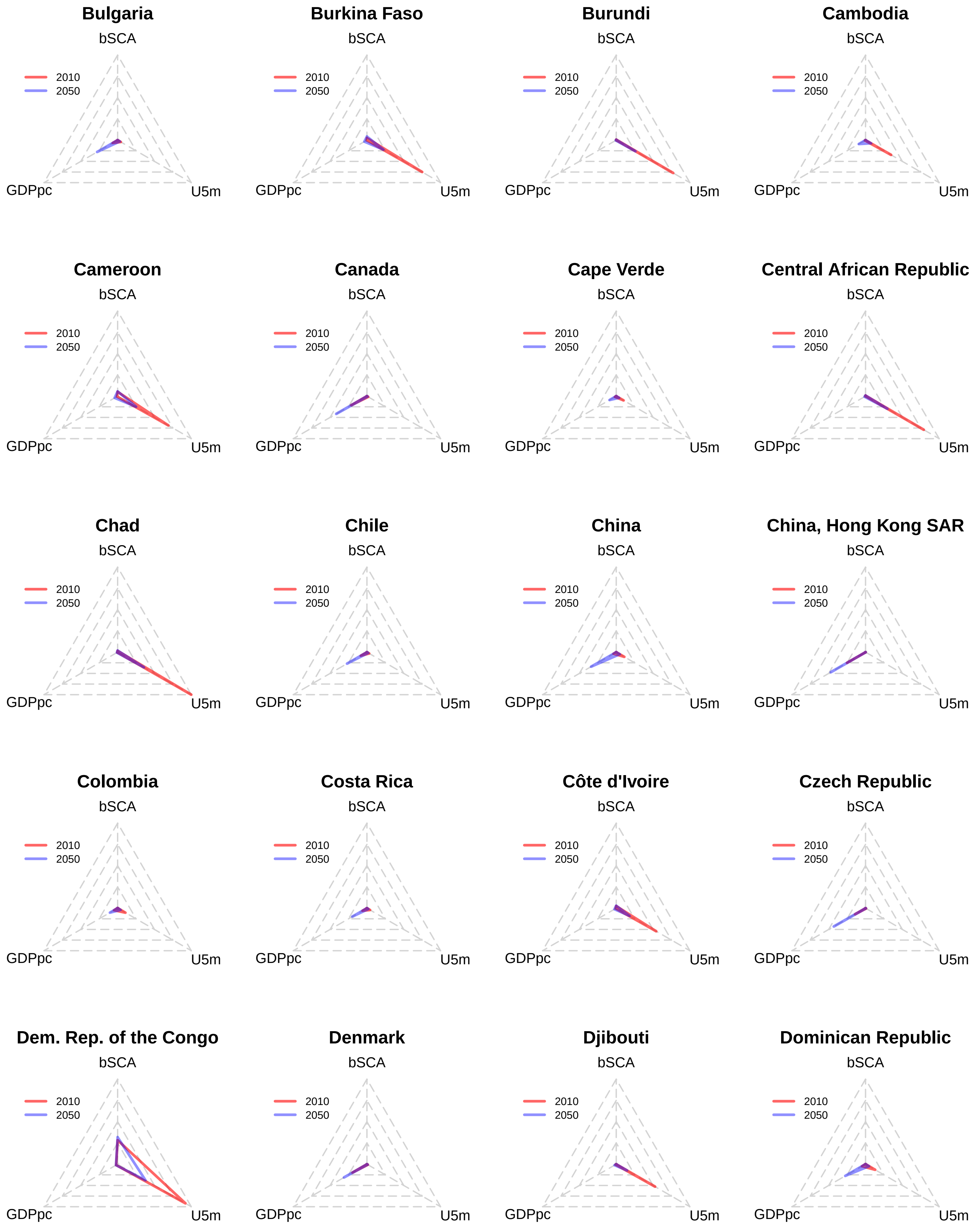
Bulgaria
Burkina Faso
Burundi
Cambodia
Cameroon
Canada
Cape Verde
Central African Republic
Chad
Chile
China
China, Hong Kong SAR
Colombia
Costa Rica
Côte d'Ivoire
Czech Republic
Dem. Rep. of the Congo
Denmark
Djibouti
Dominican Republic
bSCA
GDPpc
U5m
2010
2050

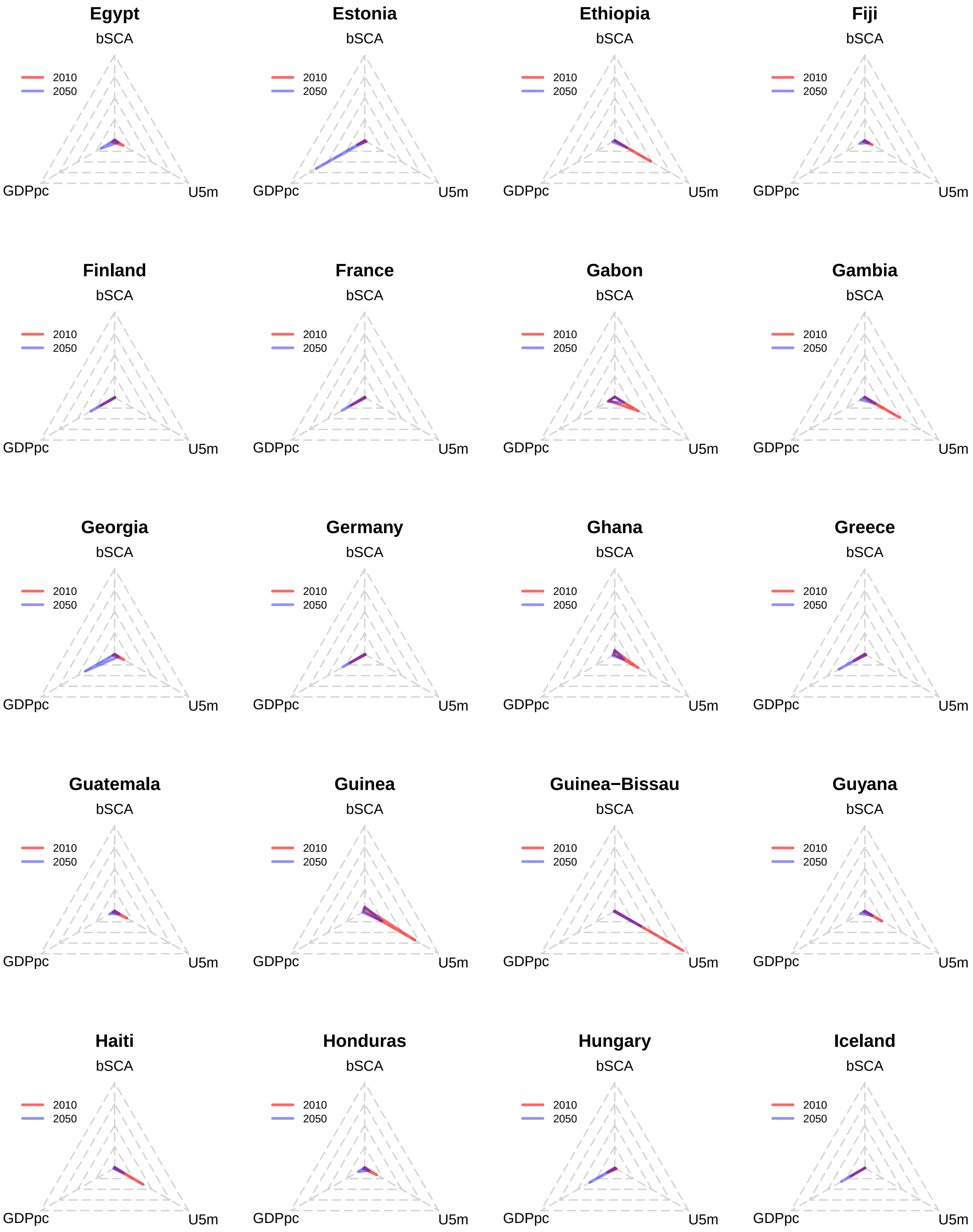

Egypt
bSCA
2010
2050
GDPpc
U5m
Estonia
bSCA
2010
2050
GDPpc
U5m
Ethiopia
bSCA
2010
2050
GDPpc
U5m
Fiji
bSCA
2010
2050
GDPpc
U5m
Finland
bSCA
2010
2050
GDPpc
U5m
France
bSCA
2010
2050
GDPpc
U5m
Gabon
bSCA
2010
2050
GDPpc
U5m
Gambia
bSCA
2010
2050
GDPpc
U5m
Georgia
bSCA
2010
2050
GDPpc
U5m
Germany
bSCA
2010
2050
GDPpc
U5m
Ghana
bSCA
2010
2050
GDPpc
U5m
Greece
bSCA
2010
2050
GDPpc
U5m
Guatemala
bSCA
2010
2050
GDPpc
U5m
Guinea
bSCA
2010
2050
GDPpc
U5m
Guinea−Bissau
bSCA
2010
2050
GDPpc
U5m
Guyana
bSCA
2010
2050
GDPpc
U5m
Haiti
bSCA
2010
2050
GDPpc
U5m
Honduras
bSCA
2010
2050
GDPpc
U5m
Hungary
bSCA
2010
2050
GDPpc
U5m
Iceland
bSCA
2010
2050
GDPpc
U5m

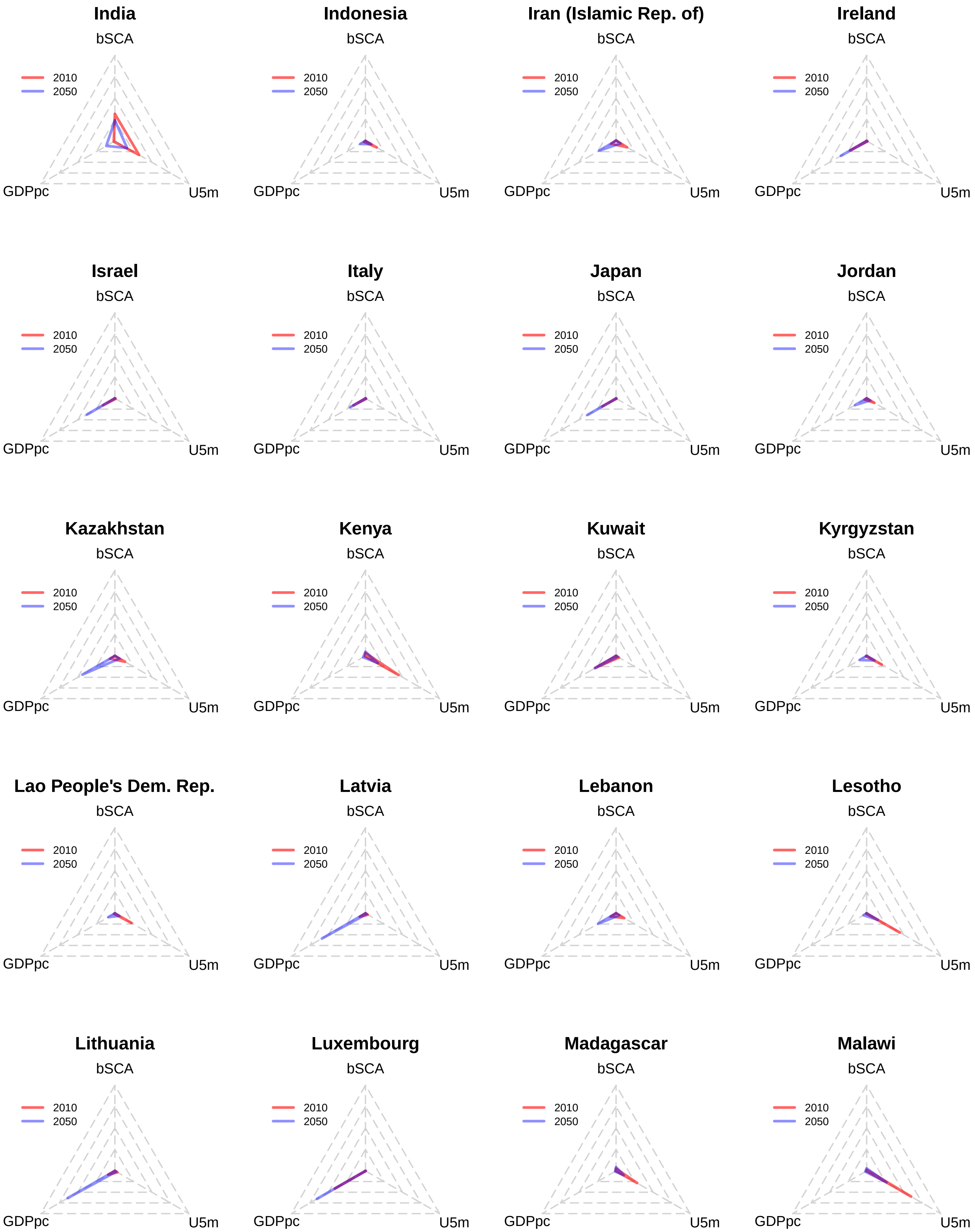
India
bSCA
2010
2050
GDPpc
U5m
Indonesia
bSCA
2010
2050
GDPpc
U5m
Iran (Islamic Rep. of)
bSCA
2010
2050
GDPpc
U5m
Ireland
bSCA
2010
2050
GDPpc
U5m
Israel
bSCA
2010
2050
GDPpc
U5m
Italy
bSCA
2010
2050
GDPpc
U5m
Japan
bSCA
2010
2050
GDPpc
U5m
Jordan
bSCA
2010
2050
GDPpc
U5m
Kazakhstan
bSCA
2010
2050
GDPpc
U5m
Kenya
bSCA
2010
2050
GDPpc
U5m
Kuwait
bSCA
2010
2050
GDPpc
U5m
Kyrgyzstan
bSCA
2010
2050
GDPpc
U5m
Lao People's Dem. Rep.
bSCA
2010
2050
GDPpc
U5m
Latvia
bSCA
2010
2050
GDPpc
U5m
Lebanon
bSCA
2010
2050
GDPpc
U5m
Lesotho
bSCA
2010
2050
GDPpc
U5m
Lithuania
bSCA
2010
2050
GDPpc
U5m
Luxembourg
bSCA
2010
2050
GDPpc
U5m
Madagascar
bSCA
2010
2050
GDPpc
U5m
Malawi
bSCA
2010
2050
GDPpc
U5m

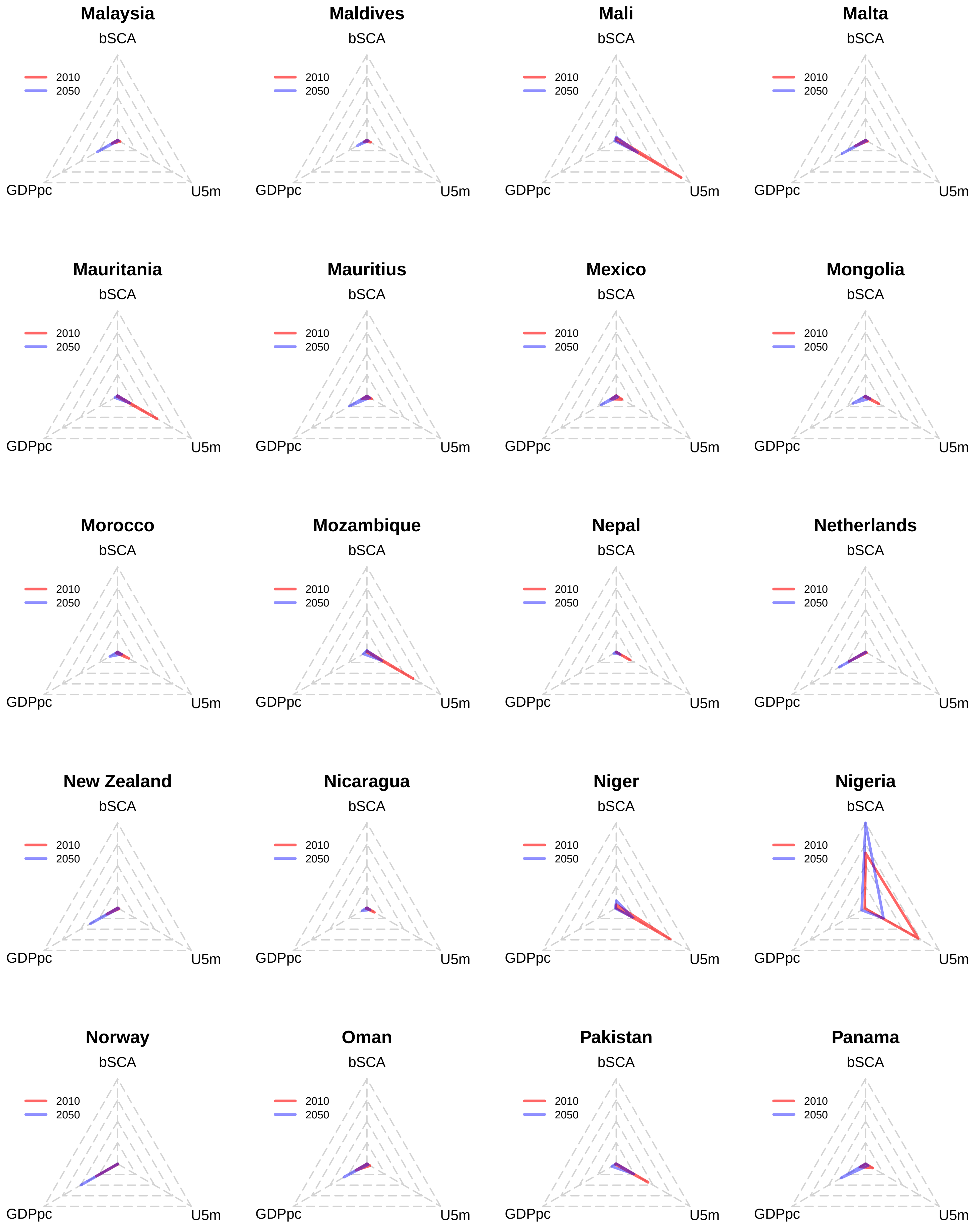
Malaysia
bSCA
2010
2050
GDPpc
U5m
Maldives
bSCA
2010
2050
GDPpc
U5m
Mali
bSCA
2010
2050
GDPpc
U5m
Malta
bSCA
2010
2050
GDPpc
U5m
Mauritania
bSCA
2010
2050
GDPpc
U5m
Mauritius
bSCA
2010
2050
GDPpc
U5m
Mexico
bSCA
2010
2050
GDPpc
U5m
Mongolia
bSCA
2010
2050
GDPpc
U5m
Morocco
bSCA
2010
2050
GDPpc
U5m
Mozambique
bSCA
2010
2050
GDPpc
U5m
Nepal
bSCA
2010
2050
GDPpc
U5m
Netherlands
bSCA
2010
2050
GDPpc
U5m
New Zealand
bSCA
2010
2050
GDPpc
U5m
Nicaragua
bSCA
2010
2050
GDPpc
U5m
Niger
bSCA
2010
2050
GDPpc
U5m
Nigeria
bSCA
2010
2050
GDPpc
U5m
Norway
bSCA
2010
2050
GDPpc
U5m
Oman
bSCA
2010
2050
GDPpc
U5m
Pakistan
bSCA
2010
2050
GDPpc
U5m
Panama
bSCA
2010
2050
GDPpc
U5m

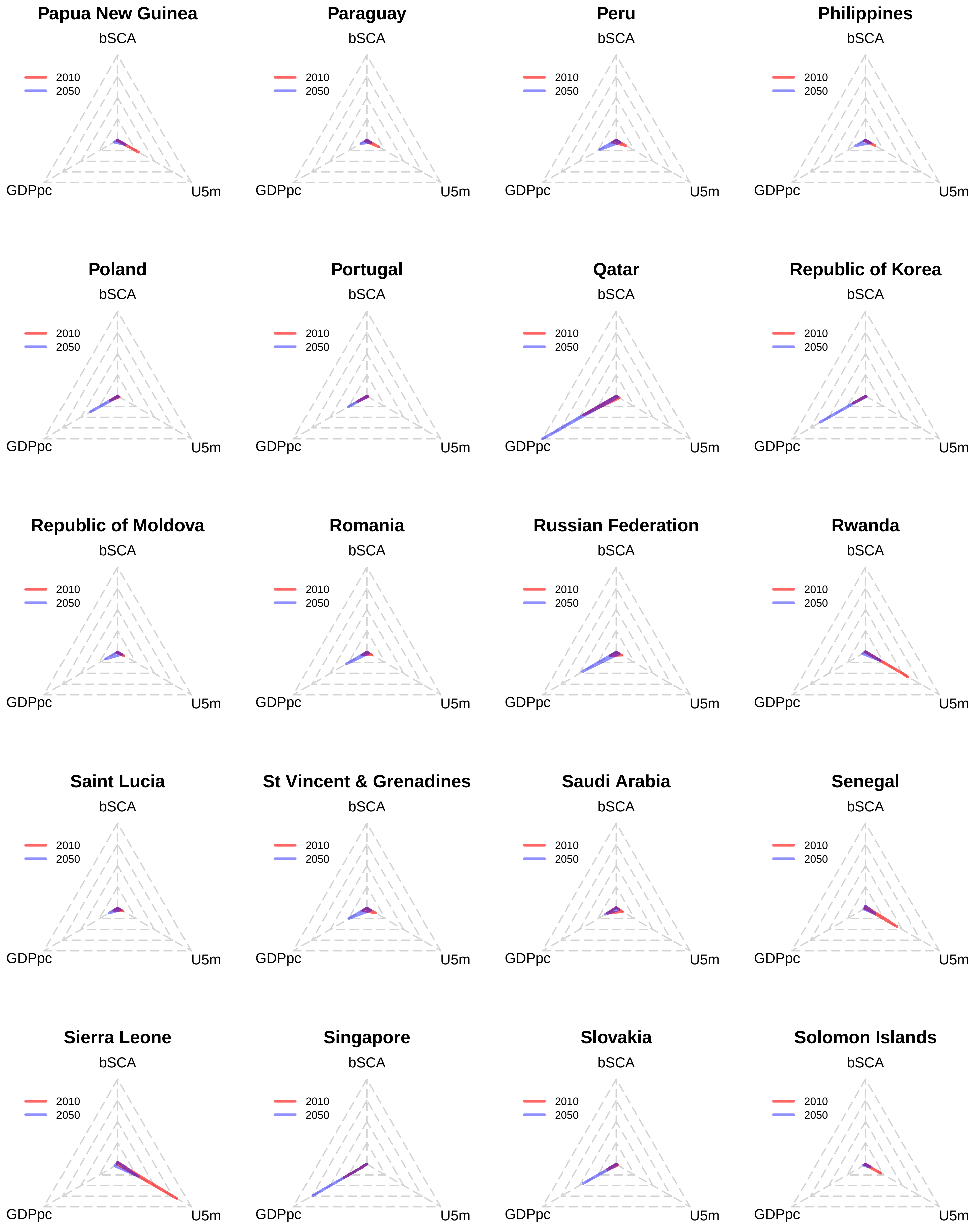
Papua New Guinea
bSCA
2010
2050
GDPpc
U5m
Paraguay
bSCA
2010
2050
GDPpc
U5m
Peru
bSCA
2010
2050
GDPpc
U5m
Philippines
bSCA
2010
2050
GDPpc
U5m
Poland
bSCA
2010
2050
GDPpc
U5m
Portugal
bSCA
2010
2050
GDPpc
U5m
Qatar
bSCA
2010
2050
GDPpc
U5m
Republic of Korea
bSCA
2010
2050
GDPpc
U5m
Republic of Moldova
bSCA
2010
2050
GDPpc
U5m
Romania
bSCA
2010
2050
GDPpc
U5m
Russian Federation
bSCA
2010
2050
GDPpc
U5m
Rwanda
bSCA
2010
2050
GDPpc
U5m
Saint Lucia
bSCA
2010
2050
GDPpc
U5m
St Vincent & Grenadines
bSCA
2010
2050
GDPpc
U5m
Saudi Arabia
bSCA
2010
2050
GDPpc
U5m
Senegal
bSCA
2010
2050
GDPpc
U5m
Sierra Leone
bSCA
2010
2050
GDPpc
U5m
Singapore
bSCA
2010
2050
GDPpc
U5m
Slovakia
bSCA
2010
2050
GDPpc
U5m
Solomon Islands
bSCA
2010
2050
GDPpc
U5m

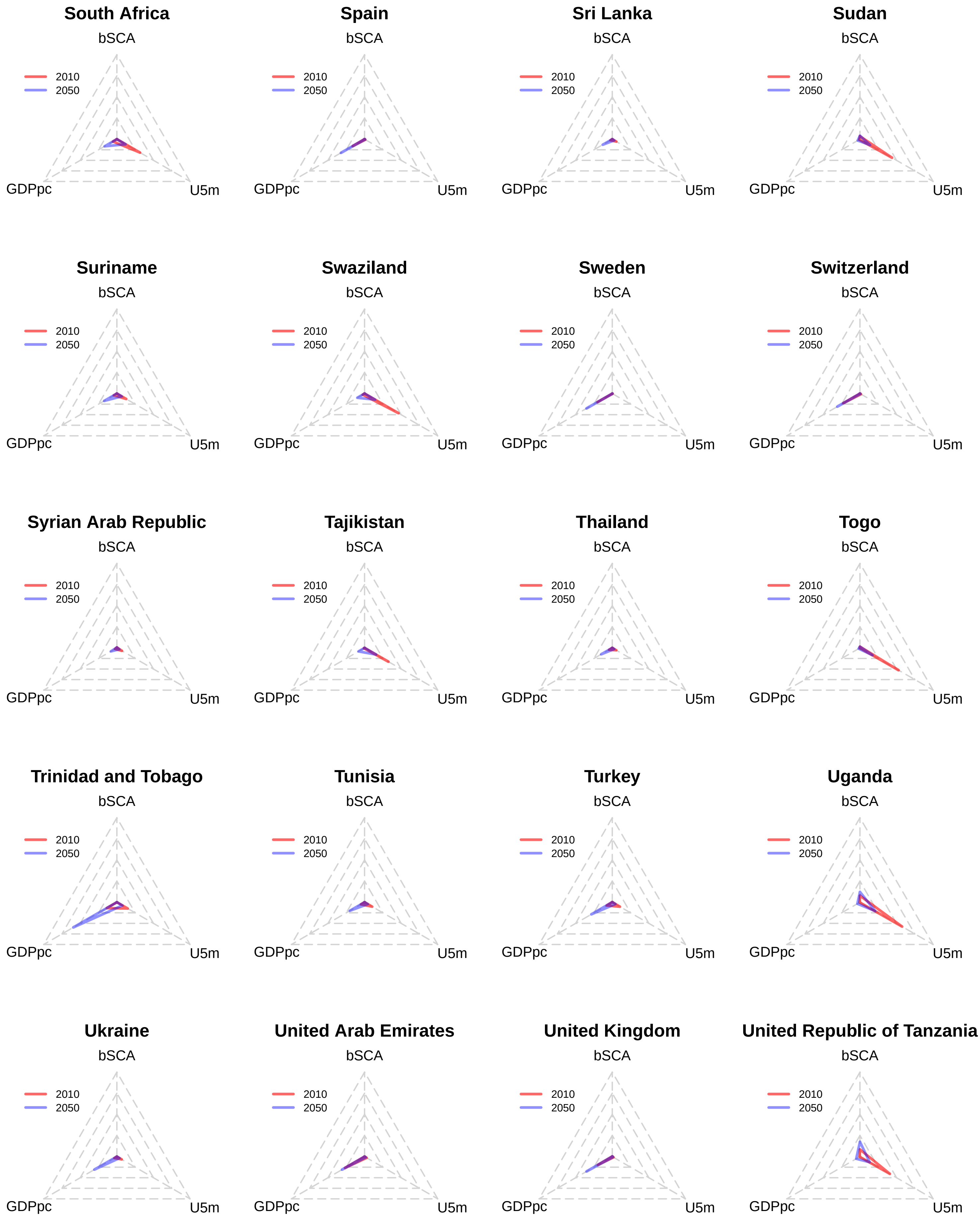
South Africa
bSCA
2010
2050
GDPpc
U5m
Spain
bSCA
2010
2050
GDPpc
U5m
Sri Lanka
bSCA
2010
2050
GDPpc
U5m
Sudan
bSCA
2010
2050
GDPpc
U5m
Suriname
bSCA
2010
2050
GDPpc
U5m
Swaziland
bSCA
2010
2050
GDPpc
U5m
Sweden
bSCA
2010
2050
GDPpc
U5m
Switzerland
bSCA
2010
2050
GDPpc
U5m
Syrian Arab Republic
bSCA
2010
2050
GDPpc
U5m
Tajikistan
bSCA
2010
2050
GDPpc
U5m
Thailand
bSCA
2010
2050
GDPpc
U5m
Togo
bSCA
2010
2050
GDPpc
U5m
Trinidad and Tobago
bSCA
2010
2050
GDPpc
U5m
Tunisia
bSCA
2010
2050
GDPpc
U5m
Turkey
bSCA
2010
2050
GDPpc
U5m
Uganda
bSCA
2010
2050
GDPpc
U5m
Ukraine
bSCA
2010
2050
GDPpc
U5m
United Arab Emirates
bSCA
2010
2050
GDPpc
U5m
United Kingdom
bSCA
2010
2050
GDPpc
U5m
United Republic of Tanzania
bSCA
2010
2050
GDPpc
U5m

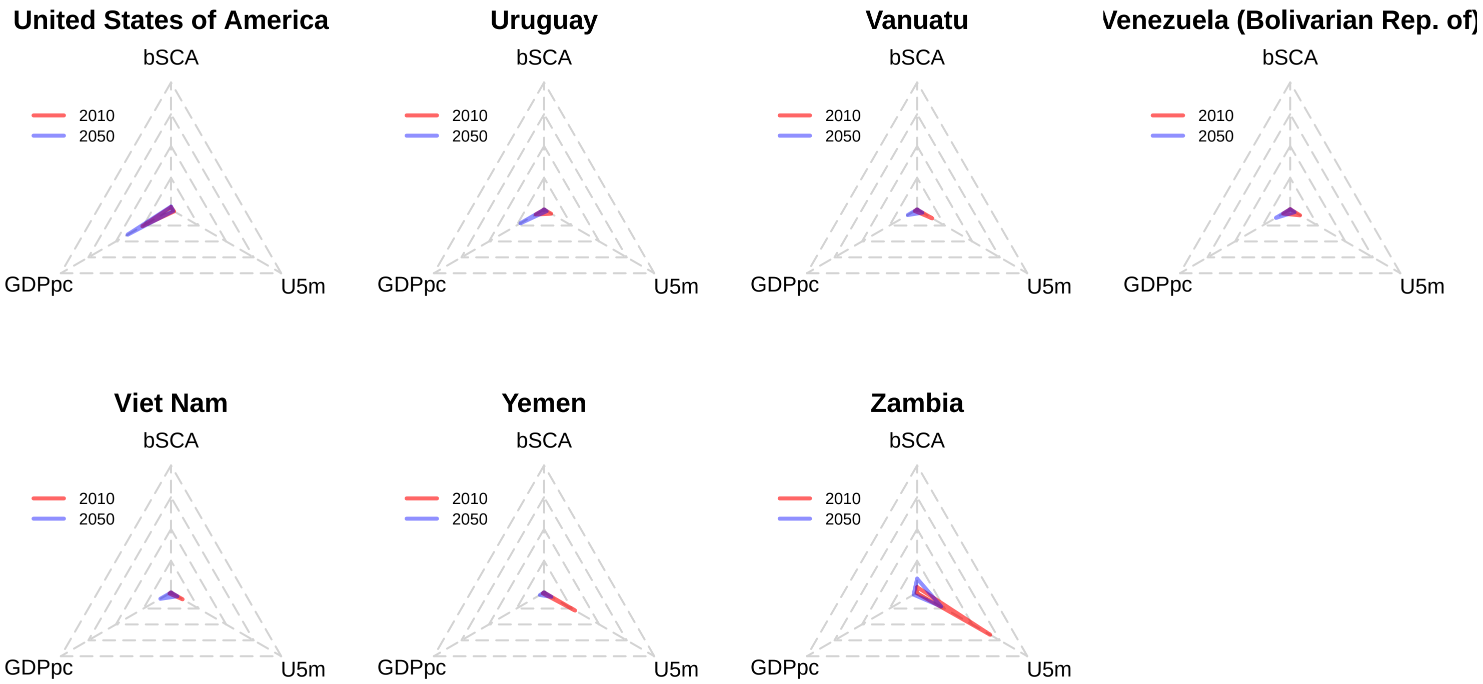
United States of America
bSCA
GDPpc
U5m
2010
2050
Uruguay
bSCA
GDPpc
U5m
2010
2050
Vanuatu
bSCA
GDPpc
U5m
2010
2050
Venezuela (Bolivarian Rep. of)
bSCA
GDPpc
U5m
2010
2050
Viet Nam
bSCA
GDPpc
U5m
2010
2050
Yemen
bSCA
GDPpc
U5m
2010
2050
Zambia
bSCA
GDPpc
U5m
2010
2050
