# Supplementary material for: Global Burden of Sickle Cell Anaemia in Children under Five, 2010–2050: Modelling Based on Demographics, Excess Mortality, and Interventions
Source: PLoS Med. 2013 Jul 16;10(7):e1001484. doi: 10.1371/journal.pmed.1001484 (PMC3712914; doi:10.1371/journal.pmed.1001484)
Supplement: Table S1 — Worked example of our model approach for Nigeria (GNIpc: US$1,180; middle-low-income level). (PDF) [file pmed.1001484.s002.pdf]

Table S1: Worked example of our model approach for Nigeria (GNI_{pc}: $1180 < $12,275; medium low income level)

			2015	2015-2020	2020-2025	2025-2030	2030-2035	2035-2040	2040-2045	2045-2050	2050	2015-2050[4]
**Input data**	**Births [A]**[1]	**Medium-fertility variant**	7,250,554	36,252,771	38,443,426	40,813,363	43,347,757	45,838,992	48,068,462	49,902,151	10,282,407	302,666,922
		**Low-fertility variant**	6,694,541	33,472,703	34,487,283	36,186,246	37,674,694	38,650,141	39,048,441	39,043,871	7,771,881	258,563,379
		**High-fertility variant**	7,806,568	39,032,838	42,399,568	45,467,753	49,158,541	53,414,858	57,860,095	61,991,071	13,140,721	349,324,724
	**SCA frequency [B]**[2]	**Median**	0.01370	0.01370	0.01370	0.01370	0.01370	0.01370	0.01370	0.01370	0.01370	0.01370
		**IQR lower bound (25%)**	0.01229	0.01229	0.01229	0.01229	0.01229	0.01229	0.01229	0.01229	0.01229	0.01229
		**IQR higher bound (75%)**	0.01527	0.01527	0.01527	0.01527	0.01527	0.01527	0.01527	0.01527	0.01527	0.01527
	**Under-five mortality (per thousand life births) [C]**[3]		141	125	111	98	86	75	65	57	57	
**Newborns with SCA [D] = [A]*[B]**		**Medium**	99,310	496,549	526,554	559,014	593,727	627,850	658,386	683,502	140,837	4,145,582
		**Low**	82,251	411,254	423,720	444,594	462,881	474,866	479,759	479,703	95,487	3,176,777
		**High**	119,174	595,869	647,265	694,104	750,447	815,423	883,283	946,346	200,604	5,332,738
**Children under 5 with SCA surviving**	**Scenario 1 (90% excess mortality) $[X_1]$**[5]	**Medium**	8,685	43,426	46,801	50,420	54,264	58,065	61,531	64,467	13,387	378,975
		**Low**	7,193	35,967	37,661	40,100	42,305	43,917	44,837	45,245	9,077	290,032
		**High**	10,423	52,113	57,531	62,604	68,587	75,412	82,549	89,259	19,069	488,055
	**Scenario 2 (50% excess mortality) $[X_2]$**[6]	**Medium**	43,426	217,132	234,007	252,099	271,318	290,325	307,656	322,337	66,937	1,894,873
		**Low**	35,967	179,834	188,306	200,499	211,525	219,583	224,185	226,227	45,383	1,450,159
		**High**	52,113	260,563	287,653	313,020	342,935	377,061	412,747	446,294	95,343	2,440,273
	**Scenario 3 (10% excess mortality) $[X_3]$**[7]	**Medium**	78,167	390,837	421,212	453,778	488,372	522,585	553,780	580,207	120,486	3,410,772
		**Low**	64,740	323,701	338,951	360,897	380,744	395,250	403,534	407,208	81,690	2,610,286
		**High**	93,803	469,013	517,775	563,437	617,282	678,710	742,945	803,329	171,617	4,392,491
	**Scenario 4 (5% excess mortality) $[X_4]$**[8]	**Medium**	82,510	412,550	444,613	478,988	515,504	551,617	584,546	612,441	127,179	3,600,260
		**Low**	68,337	341,685	357,782	380,947	401,897	417,208	425,952	429,830	86,228	2,755,302
		**High**	99,014	495,070	546,540	594,739	651,576	716,416	784,220	847,959	181,151	4,636,519
**Lives of newborns with SCA saved**	**Sc2 vs Sc1 $[X_2]$-$[X_1]$**	**Medium**	34,741	173,705	187,205	201,679	217,054	232,260	246,125	257,870	53,549	1,515,899
		**Low**	28,773	143,867	150,645	160,399	169,220	175,667	179,348	180,981	36,306	1,160,127
		**High**	41,690	208,450	230,122	250,416	274,348	301,649	330,198	357,035	76,274	1,952,218
	**Sc3 vs Sc1 $[X_3]$-$[X_1]$**	**Medium**	69,482	347,411	374,411	403,358	434,109	464,520	492,249	515,740	107,099	3,031,798
		**Low**	57,547	287,735	301,290	320,798	338,439	351,333	358,697	361,962	72,613	2,320,254
		**High**	83,380	416,901	460,244	500,833	548,696	603,298	660,396	714,071	152,549	3,904,437
	**Sc4 vs Sc1 $[X_4]$-$[X_1]$**	**Medium**	73,825	369,124	397,811	428,568	461,241	493,552	523,015	547,974	113,792	3,221,285
		**Low**	61,144	305,718	320,120	340,848	359,592	373,292	381,115	384,585	77,151	2,465,270
		**High**	88,591	442,957	489,009	532,135	582,989	641,004	701,670	758,700	162,083	4,148,464

[1] From the 2010 Revision of the United Nations World Population Prospects (http://esa.un.org/unpd/wpp/index.htm).

[2] SCA: Sickle cell anaemia. Output from the Bayesian geostatistical model described in Piel et al 2013, for 2010 (http://www.thelancet.com/journals/lancet/article/PIIS0140-6736%2812%2961229-X/).

[3] From the 2010 Revision of the United Nations World Population Prospects (http://esa.un.org/unpd/wpp/index.htm).

[4] Sum of estimates between 2015 and 2050 (based on five-year intervals).

[5] Calculated as: [D]*(1-([C]/1000)*(1-0.9).

[6] Calculated as: [D]*(1-([C]/1000)*(1-0.5).

[7] Calculated as: [D]*(1-([C]/1000)*(1-0.1).

[8] Calculated as: [D]*(1-([C]/1000)*(1-0.05).
