# Supplementary material for: Global Burden of Sickle Cell Anaemia in Children under Five, 2010–2050: Modelling Based on Demographics, Excess Mortality, and Interventions
Source: PLoS Med. 2013 Jul 16;10(7):e1001484. doi: 10.1371/journal.pmed.1001484 (PMC3712914; doi:10.1371/journal.pmed.1001484)
Supplement: Table S2 — National, regional, and global SCA frequency (median and interquartile range), projected births (in thousands) with CI, and estimated number of newborns with SCA with CI. (PDF) [file pmed.1001484.s003.pdf]

Table S2 National, regional and global SCA frequency (median and interquartile range (IQR)), projected births (in thousands) with confidence interval (CI) and estimated number of newborns with SCA with CI.

Country/Region	SCA frequency [IQR][1]	Births 2010 [CI][2]	Births 2050 [CI][2]	Births 2010-2050 [CI][3]	Newborns with SCA 2010 [CI][4]	Newborns with SCA 2050 [CI][4]	Newborns with SCA 2010-2050 [CI][5]	Rank[6]
Afghanistan	0.000 [0.000-0.000]	1,440 [1,380-1,501]	1,724 [1,251-2,269]	66,324 [56,968-76,197]	0 [0-10]	1 [0-15]	21 [0-511]	*50*
Albania	0.001 [0.000-0.002]	41 [35-48]	24 [11-44]	1,311 [869-1,802]	30 [12-69]	18 [4-62]	964 [301-2,579]	*159*
Algeria	0.000 [0.000-0.001]	703 [621-786]	481 [257-774]	22,777 [16,352-29,739]	197 [105-384]	135 [43-378]	6,388 [2,760-14,523]	*173*
Angola	0.011 [0.003-0.027]	817 [777-857]	853 [574-1,186]	35,123 [29,226-41,447]	9,017 [6,030-13,267]	9,417 [4,456-18,371]	387,648 [226,743-641,809]	24
Argentina	0.000 [0.000-0.000]	692 [612-772]	582 [324-918]	25,600 [18,694-33,232]	5 [1-24]	4 [0-29]	167 [17-1,051]	*136*
Armenia	0.000 [0.000-0.000]	47 [40-53]	29 [15-48]	1,444 [1,016-1,922]	0 [0-0]	0 [0-0]	0 [0-1]	*98*
Aruba	0.001 [0.000-0.002]	1 [1-1]	1 [1-2]	46 [32-61]	1 [0-2]	1 [0-2]	33 [10-88]	*122*
Australia	0.000 [0.000-0.000]	312 [272-352]	383 [234-567]	13,776 [10,244-17,557]	6 [1-22]	7 [1-35]	248 [46-1,086]	*47*
Austria	0.000 [0.000-0.000]	73 [59-86]	78 [42-125]	2,971 [1,994-4,028]	1 [0-12]	1 [0-18]	38 [1-567]	*51*
Azerbaijan	0.000 [0.000-0.000]	184 [163-206]	141 [83-214]	6,200 [4,622-7,945]	0 [0-0]	0 [0-0]	0 [0-14]	*102*
Bahamas	0.002 [0.001-0.006]	5 [5-6]	5 [3-7]	202 [145-263]	8 [2-27]	7 [1-34]	308 [69-1,185]	*137*
Bahrain	0.004 [0.002-0.011]	24 [21-26]	17 [10-27]	785 [582-1,007]	97 [47-178]	70 [21-181]	3,202 [1,276-6,818]	*167*
Bangladesh	0.000 [0.000-0.000]	2,998 [2,650-3,345]	1,907 [939-3,237]	98,644 [69,313-131,635]	15 [1-106]	9 [1-102]	487 [38-4,165]	*152*
Barbados	0.002 [0.001-0.007]	3 [3-3]	3 [1-4]	111 [78-149]	5 [1-19]	4 [0-23]	177 [22-813]	*133*
Belarus	0.000 [0.000-0.000]	106 [88-124]	82 [43-134]	3,632 [2,511-4,875]	0 [0-0]	0 [0-0]	0 [0-0]	*91*
Belgium	0.000 [0.000-0.001]	122 [106-139]	135 [81-202]	5,056 [3,733-6,466]	29 [15-55]	32 [12-80]	1,200 [534-2,575]	*45*
Belize	0.006 [0.002-0.016]	8 [7-9]	7 [4-11]	306 [229-391]	49 [29-80]	43 [16-100]	1,924 [941-3,658]	*156*
Benin	0.013 [0.002-0.028]	363 [345-381]	518 [377-681]	17,691 [15,028-20,517]	4,672 [3,723-5,785]	6,661 [4,063-10,335]	227,640 [162,084-311,549]	11
Bhutan	0.000 [0.000-0.000]	15 [13-16]	9 [5-16]	482 [341-639]	0 [0-0]	0 [0-0]	0 [0-5]	*109*
Bolivia (Plurinational State of)	0.000 [0.000-0.000]	266 [246-287]	263 [164-385]	10,866 [8,545-13,396]	0 [0-0]	0 [0-0]	0 [0-12]	*99*
Bosnia and Herzegovina	0.000 [0.000-0.000]	31 [24-38]	22 [10-39]	1,043 [649-1,483]	1 [0-10]	1 [0-10]	30 [1-382]	*127*
Botswana	0.000 [0.000-0.000]	47 [43-52]	35 [19-54]	1,665 [1,247-2,123]	5 [1-17]	3 [1-18]	160 [35-692]	*139*
Brazil	0.001 [0.000-0.003]	2,978 [2,565-3,392]	2,075 [1,011-3,572]	100,805 [68,307-137,935]	3,295 [2,022-5,288]	2,296 [797-5,569]	111,537 [53,858-215,030]	193
Brunei Darussalam	0.000 [0.000-0.000]	8 [7-9]	7 [4-11]	287 [202-380]	0 [0-0]	0 [0-0]	0 [0-0]	*75*
Bulgaria	0.000 [0.000-0.001]	73 [62-85]	52 [27-85]	2,433 [1,702-3,244]	12 [1-73]	12 [1-73]	567 [87-2,785]	*151*
Burkina Faso	0.005 [0.001-0.010]	755 [722-788]	1,306 [1,001-1,652]	40,409 [35,124-45,985]	3,423 [2,810-4,126]	5,924 [3,897-8,651]	183,238 [136,678-240,844]	7
Burundi	0.003 [0.001-0.008]	294 [276-312]	216 [135-315]	10,396 [8,389-12,547]	920 [560-1,453]	677 [275-1,467]	32,578 [17,058-58,455]	190
Cambodia	0.000 [0.000-0.000]	315 [282-347]	208 [109-338]	10,444 [7,600-13,561]	0 [0-0]	0 [0-0]	0 [0-0]	*81*
Cameroon	0.010 [0.001-0.021]	722 [680-764]	800 [544-1,107]	30,989 [25,546-36,847]	7,172 [5,927-8,619]	7,941 [4,738-12,478]	307,667 [222,606-415,425]	17
Canada	0.000 [0.000-0.000]	393 [335-451]	488 [290-738]	17,139 [12,310-22,325]	46 [15-122]	57 [13-200]	2,005 [560-6,057]	*41*
Cape Verde	0.002 [0.001-0.004]	10 [9-11]	6 [3-11]	328 [234-433]	17 [9-30]	11 [3-29]	566 [243-1,173]	*157*
Central African Republic	0.006 [0.002-0.016]	159 [150-169]	167 [111-233]	6,763 [5,569-8,047]	1,001 [617-1,558]	1,045 [457-2,152]	42,455 [22,849-74,386]	34
Chad	0.004 [0.001-0.010]	521 [498-543]	667 [484-878]	24,427 [20,926-28,143]	2,132 [1,455-3,034]	2,732 [1,415-4,904]	100,014 [61,140-157,110]	20
Chile	0.000 [0.000-0.000]	245 [212-279]	197 [103-323]	8,830 [6,156-11,817]	0 [0-0]	0 [0-0]	0 [0-0]	*83*
China	0.000 [0.000-0.000]	16,170 [13,576-18,764]	11,140 [5,759-18,347]	529,129 [360,082-716,740]	0 [0-1]	0 [0-1]	1 [0-36]	*111*
China, Hong Kong SAR	0.000 [0.000-0.000]	64 [50-78]	93 [53-143]	2,916 [1,937-3,968]	0 [0-0]	0 [0-0]	0 [0-0]	*66*
China, Macao SAR	0.000 [0.000-0.000]	6 [5-7]	8 [4-12]	263 [174-360]	0 [0-0]	0 [0-0]	0 [0-0]	*67*
Colombia	0.000 [0.000-0.001]	903 [804-1,001]	695 [378-1,115]	32,344 [23,528-42,142]	332 [116-848]	255 [54-945]	11,878 [3,383-35,710]	*179*
Comoros	0.001 [0.001-0.003]	28 [26-29]	40 [29-53]	1,345 [1,137-1,567]	28 [11-66]	40 [12-120]	1,337 [479-3,537]	*40*
Congo	0.012 [0.003-0.027]	148 [139-156]	200 [140-271]	6,955 [5,787-8,211]	1,772 [1,306-2,409]	2,396 [1,310-4,178]	83,402 [54,180-126,738]	19
Costa Rica	0.001 [0.001-0.003]	74 [64-84]	55 [28-93]	2,573 [1,754-3,497]	62 [16-205]	47 [7-227]	2,174 [443-8,524]	*162*
Côte d'Ivoire	0.005 [0.001-0.012]	688 [647-729]	818 [558-1,126]	30,837 [25,416-36,640]	3,447 [2,408-4,886]	4,097 [2,076-7,553]	154,537 [94,557-245,693]	18
Croatia	0.000 [0.000-0.000]	43 [36-50]	38 [20-61]	1,593 [1,103-2,134]	1 [0-11]	1 [0-13]	42 [2-469]	*123*
Cuba	0.002 [0.001-0.007]	108 [89-126]	79 [38-136]	3,671 [2,406-5,114]	240 [92-545]	176 [39-589]	8,177 [2,492-22,087]	176
Cyprus	0.000 [0.000-0.001]	13 [11-15]	13 [7-21]	520 [352-704]	2 [1-9]	2 [0-12]	92 [17-405]	*117*
Czech Republic	0.000 [0.000-0.000]	115 [96-135]	114 [65-176]	4,310 [3,051-5,665]	0 [0-6]	0 [0-7]	6 [0-235]	*107*
Dem. People's Republic of Korea	0.000 [0.000-0.000]	350 [306-394]	316 [174-501]	13,449 [9,743-17,505]	0 [0-0]	0 [0-0]	0 [0-0]	*71*
Democratic Republic of the Congo	0.013 [0.002-0.029]	2,970 [2,834-3,105]	3,337 [2,353-4,489]	131,599 [111,455-153,071]	39,743 [32,593-48,788]	44,663 [27,062-70,542]	1,761,226 [1,281,666-2,405,181]	6
Denmark	0.000 [0.000-0.000]	63 [55-72]	68 [40-104]	2,696 [1,990-3,453]	0 [0-1]	0 [0-1]	1 [0-45]	*54*
Djibouti	0.000 [0.000-0.000]	26 [25-28]	27 [18-39]	1,096 [880-1,327]	0 [0-0]	0 [0-0]	0 [0-4]	*56*
Dominican Republic	0.001 [0.001-0.004]	214 [192-235]	150 [78-247]	7,493 [5,493-9,753]	247 [70-740]	173 [29-778]	8,655 [2,012-30,674]	177
Ecuador	0.000 [0.000-0.000]	296 [265-327]	217 [116-352]	10,513 [7,678-13,681]	0 [0-3]	0 [0-4]	4 [0-141]	*116*
Egypt	0.001 [0.000-0.002]	1,888 [1,709-2,067]	1,529 [875-2,367]	69,793 [52,723-88,480]	1,199 [359-3,397]	971 [184-3,890]	44,320 [11,067-145,410]	189
El Salvador	0.001 [0.000-0.002]	125 [111-140]	82 [42-138]	4,224 [3,010-5,586]	81 [30-190]	53 [11-187]	2,734 [800-7,577]	*168*
Equatorial Guinea	0.015 [0.004-0.035]	27 [26-28]	31 [21-42]	1,198 [1,000-1,411]	418 [306-562]	478 [253-840]	18,531 [11,921-27,962]	32

Country/Region	SCA frequency [IQR][1]	Births 2010 [CI][2]	Births 2050 [CI][2]	Births 2010-2050 [CI][3]	Newborns with SCA 2010 [CI][4]	Newborns with SCA 2050 [CI][4]	Newborns with SCA 2010-2050 [CI][5]	Rank[6]
Eritrea	0.000 [0.000-0.000]	195 [183-206]	213 [143-296]	8,302 [6,817-9,887]	9 [2-40]	10 [2-57]	402 [72-1,904]	*48*
Estonia	0.000 [0.000-0.000]	16 [14-19]	14 [8-22]	571 [413-742]	0 [0-0]	0 [0-0]	0 [0-0]	*82*
Ethiopia	0.000 [0.000-0.000]	2,621 [2,450-2,791]	2,067 [1,227-3,112]	97,974 [77,191-120,314]	141 [58-321]	111 [29-358]	5,270 [1,815-13,856]	*169*
Fiji	0.000 [0.000-0.000]	18 [16-20]	13 [8-20]	618 [472-776]	0 [0-0]	0 [0-0]	0 [0-0]	*73*
Finland	0.000 [0.000-0.000]	61 [53-70]	64 [38-98]	2,485 [1,832-3,186]	0 [0-0]	0 [0-0]	0 [0-0]	*59*
France	0.001 [0.000-0.002]	791 [691-890]	859 [519-1,284]	32,633 [24,355-41,511]	530 [258-1,048]	576 [194-1,512]	21,868 [9,090-48,897]	*33*
French Guiana	0.001 [0.000-0.002]	6 [5-6]	9 [6-12]	298 [238-364]	5 [3-9]	8 [3-18]	266 [123-532]	*46*
French Polynesia	0.000 [0.000-0.000]	5 [4-5]	3 [2-5]	157 [110-208]	0 [0-0]	0 [0-0]	0 [0-0]	*78*
Gabon	0.021 [0.004-0.047]	43 [39-46]	51 [33-72]	1,897 [1,508-2,319]	911 [685-1,204]	1,085 [580-1,889]	40,437 [26,231-60,571]	27
Gambia	0.006 [0.001-0.014]	68 [65-72]	87 [61-118]	3,185 [2,664-3,739]	443 [360-543]	564 [339-888]	20,616 [14,807-28,155]	28
Georgia	0.000 [0.000-0.000]	49 [41-57]	28 [13-47]	1,425 [962-1,947]	0 [0-0]	0 [0-0]	0 [0-0]	*94*
Germany	0.000 [0.000-0.000]	709 [588-831]	755 [422-1,184]	28,699 [20,057-38,052]	84 [18-311]	90 [13-443]	3,410 [629-14,228]	*42*
Ghana	0.007 [0.001-0.016]	783 [734-832]	923 [627-1,277]	34,717 [28,466-41,406]	5,815 [4,903-6,831]	6,855 [4,184-10,477]	257,716 [190,070-339,744]	14
Greece	0.002 [0.001-0.005]	116 [97-134]	115 [66-179]	4,453 [3,151-5,859]	211 [121-383]	211 [83-508]	8,126 [3,950-16,670]	129
Grenada	0.003 [0.002-0.010]	2 [2-2]	1 [0-2]	59 [43-77]	6 [1-16]	3 [0-11]	163 [33-530]	*147*
Guadeloupe	0.002 [0.001-0.006]	6 [5-7]	5 [3-8]	224 [164-289]	14 [7-24]	11 [3-27]	492 [218-1,010]	*146*
Guam	0.000 [0.000-0.000]	3 [3-4]	3 [2-5]	129 [96-166]	0 [0-0]	0 [0-0]	0 [0-0]	*68*
Guatemala	0.001 [0.000-0.002]	484 [452-515]	604 [401-852]	22,294 [18,019-26,934]	269 [105-649]	336 [93-1,073]	12,397 [4,179-33,924]	30
Guinea	0.013 [0.003-0.030]	401 [381-421]	528 [381-699]	18,826 [15,956-21,876]	5,402 [3,987-7,115]	7,123 [3,990-11,828]	253,763 [166,999-369,945]	13
Guinea-Bissau	0.003 [0.001-0.008]	60 [57-63]	80 [59-104]	2,779 [2,371-3,212]	186 [113-289]	247 [117-478]	8,642 [4,742-14,815]	31
Guyana	0.001 [0.001-0.005]	13 [12-15]	9 [4-14]	448 [325-584]	17 [4-59]	11 [1-57]	582 [111-2,333]	*155*
Haiti	0.004 [0.001-0.010]	266 [245-287]	200 [120-300]	9,605 [7,505-11,869]	1,104 [706-1,676]	831 [347-1,754]	39,926 [21,675-69,407]	191
Honduras	0.004 [0.002-0.011]	206 [189-223]	174 [101-266]	7,906 [6,066-9,929]	810 [354-1,675]	682 [190-1,994]	31,088 [11,383-74,488]	185
Hungary	0.000 [0.000-0.000]	100 [83-118]	98 [55-155]	3,848 [2,687-5,097]	0 [0-4]	0 [0-6]	7 [0-185]	*110*
Iceland	0.000 [0.000-0.000]	5 [4-5]	5 [3-8]	203 [153-257]	0 [0-0]	0 [0-0]	0 [0-0]	*62*
India	0.002 [0.000-0.004]	26,957 [24,302-29,613]	20,564 [11,495-32,415]	973,913 [726,643-1,248,292]	44,425 [33,692-59,143]	33,890 [15,936-64,740]	1,605,013 [1,007,436-2,493,101]	194
Indonesia	0.000 [0.000-0.000]	4,270 [3,751-4,790]	3,096 [1,663-4,991]	146,646 [104,670-192,989]	1 [0-8]	1 [0-9]	28 [1-336]	*126*
Iran (Islamic Republic of)	0.001 [0.000-0.002]	1,228 [1,034-1,421]	714 [333-1,242]	35,864 [23,412-49,798]	870 [378-1,846]	506 [122-1,613]	25,413 [8,548-64,707]	192
Iraq	0.001 [0.000-0.002]	1,170 [1,106-1,235]	1,866 [1,341-2,477]	60,676 [50,868-71,130]	687 [263-1,837]	1,095 [319-3,685]	35,597 [12,117-105,812]	23
Ireland	0.000 [0.000-0.000]	72 [63-80]	75 [47-110]	2,823 [2,137-3,554]	0 [0-5]	0 [0-6]	7 [0-202]	*52*
Israel	0.001 [0.001-0.004]	158 [145-172]	174 [112-249]	6,655 [5,266-8,143]	236 [135-381]	259 [104-550]	9,919 [4,890-18,028]	38
Italy	0.000 [0.000-0.001]	552 [458-645]	551 [314-856]	21,508 [15,173-28,306]	92 [31-278]	92 [21-369]	3,575 [1,029-12,189]	*118*
Jamaica	0.003 [0.001-0.007]	50 [44-55]	27 [13-47]	1,587 [1,153-2,078]	139 [89-212]	76 [26-180]	4,459 [2,328-8,016]	174
Japan	0.000 [0.000-0.000]	1,068 [880-1,256]	950 [514-1,520]	39,642 [27,599-52,633]	0 [0-0]	0 [0-0]	0 [0-0]	*76*
Jordan	0.001 [0.000-0.003]	154 [140-167]	118 [66-185]	5,701 [4,325-7,198]	146 [74-284]	112 [35-314]	5,407 [2,267-12,226]	171
Kazakhstan	0.000 [0.000-0.000]	340 [306-374]	314 [195-462]	12,775 [9,846-15,953]	0 [0-0]	0 [0-0]	0 [0-1]	*95*
Kenya	0.003 [0.000-0.006]	1,604 [1,517-1,690]	2,282 [1,614-3,069]	77,436 [64,694-91,116]	4,762 [3,905-5,763]	6,776 [4,154-10,464]	229,970 [166,550-310,635]	10
Kuwait	0.002 [0.002-0.008]	51 [45-56]	67 [42-98]	2,327 [1,746-2,955]	111 [31-311]	146 [28-542]	5,079 [1,184-16,331]	35
Kyrgyzstan	0.000 [0.000-0.000]	132 [119-144]	122 [75-181]	5,026 [3,872-6,294]	0 [0-0]	0 [0-0]	0 [0-0]	*86*
Lao People's Democratic Republic	0.000 [0.000-0.000]	140 [126-153]	90 [46-148]	4,707 [3,439-6,097]	0 [0-0]	0 [0-0]	0 [0-0]	*85*
Latvia	0.000 [0.000-0.000]	24 [20-29]	20 [11-33]	848 [597-1,122]	0 [0-0]	0 [0-0]	0 [0-0]	*84*
Lebanon	0.004 [0.002-0.010]	64 [55-73]	44 [22-75]	2,159 [1,479-2,908]	256 [133-442]	176 [52-451]	8,597 [3,568-17,591]	180
Lesotho	0.000 [0.000-0.000]	60 [56-65]	46 [27-70]	2,191 [1,697-2,732]	0 [0-2]	0 [0-2]	4 [0-80]	*115*
Liberia	0.004 [0.001-0.008]	161 [153-169]	231 [168-303]	7,825 [6,647-9,078]	586 [438-780]	841 [483-1,402]	28,522 [19,086-41,998]	25
Libyan Arab Jamahiriya	0.001 [0.000-0.003]	141 [126-155]	93 [51-146]	4,467 [3,284-5,726]	187 [114-292]	123 [46-274]	5,927 [2,964-10,755]	175
Lithuania	0.000 [0.000-0.000]	36 [30-42]	31 [17-50]	1,274 [892-1,691]	0 [0-0]	0 [0-0]	0 [0-0]	*87*
Luxembourg	0.000 [0.000-0.000]	6 [5-7]	8 [5-12]	291 [210-378]	0 [0-1]	0 [0-2]	16 [3-75]	*49*
Madagascar	0.005 [0.001-0.010]	771 [728-814]	1,251 [884-1,684]	40,279 [33,483-47,592]	3,736 [3,031-4,570]	6,066 [3,683-9,461]	195,261 [139,419-267,333]	8
Malawi	0.002 [0.001-0.006]	721 [691-751]	1,650 [1,296-2,048]	44,644 [39,013-50,596]	1,743 [1,018-2,975]	3,990 [1,909-8,111]	107,938 [57,481-200,387]	9
Malaysia	0.000 [0.000-0.000]	584 [527-641]	564 [340-843]	23,622 [18,096-29,539]	0 [0-0]	0 [0-0]	0 [0-0]	*92*
Maldives	0.000 [0.000-0.000]	5 [5-6]	3 [1-6]	164 [106-229]	0 [0-0]	0 [0-0]	0 [0-0]	*89*
Mali	0.004 [0.001-0.010]	746 [716-777]	1,159 [881-1,473]	38,162 [33,226-43,370]	3,353 [2,462-4,534]	5,203 [3,031-8,596]	171,400 [114,272-253,084]	12
Malta	0.001 [0.001-0.003]	4 [3-5]	4 [2-6]	146 [95-202]	3 [0-10]	2 [0-13]	96 [15-439]	*121*
Martinique	0.003 [0.001-0.007]	5 [4-5]	3 [2-6]	164 [117-216]	12 [7-22]	9 [3-22]	431 [190-872]	*148*
Mauritania	0.004 [0.002-0.011]	120 [113-127]	147 [103-199]	5,392 [4,497-6,344]	471 [246-862]	579 [225-1,352]	21,237 [9,812-43,209]	29
Mauritius	0.000 [0.000-0.000]	16 [14-19]	14 [7-22]	603 [412-814]	0 [0-0]	0 [0-0]	0 [0-2]	*96*
Mayotte	0.002 [0.002-0.008]	7 [7-8]	9 [6-12]	332 [271-398]	13 [2-46]	16 [2-75]	610 [91-2,429]	*44*

Country/Region	SCA frequency [IQR][1]	Births 2010 [CI][2]	Births 2050 [CI][2]	Births 2010-2050 [CI][3]	Newborns with SCA 2010 [CI][4]	Newborns with SCA 2050 [CI][4]	Newborns with SCA 2010-2050 [CI][5]	Rank[6]
Mexico	0.000 [0.000-0.001]	2,158 [1,916-2,401]	1,471 [756-2,441]	72,929 [52,190-96,159]	676 [395-1,145]	461 [156-1,164]	22,850 [10,761-45,849]	188
Micronesia (Fed. States of)	0.000 [0.000-0.000]	3 [3-3]	2 [1-3]	99 [80-120]	0 [0-0]	0 [0-0]	0 [0-0]	77
Mongolia	0.000 [0.000-0.000]	65 [58-72]	60 [37-89]	2,447 [1,879-3,065]	0 [0-0]	0 [0-0]	0 [0-0]	80
Montenegro	0.000 [0.000-0.001]	8 [6-9]	6 [3-10]	264 [183-352]	1 [0-4]	0 [0-5]	22 [1-179]	124
Morocco	0.000 [0.000-0.000]	613 [543-683]	418 [225-669]	20,471 [14,958-26,356]	42 [5-246]	28 [2-241]	1,388 [139-9,479]	160
Mozambique	0.002 [0.001-0.005]	900 [852-948]	1,161 [812-1,572]	41,999 [35,095-49,389]	1,742 [1,196-2,491]	2,248 [1,140-4,133]	81,289 [49,266-129,833]	22
Myanmar	0.000 [0.000-0.000]	815 [710-920]	585 [307-950]	27,542 [19,560-36,143]	0 [0-1]	0 [0-1]	2 [0-40]	114
Namibia	0.001 [0.000-0.002]	60 [55-65]	53 [32-79]	2,347 [1,822-2,913]	33 [17-64]	29 [10-78]	1,275 [565-2,854]	149
Nepal	0.000 [0.000-0.000]	724 [654-794]	561 [301-906]	26,534 [19,442-34,440]	11 [1-76]	9 [0-87]	409 [29-3,312]	145
Netherlands	0.000 [0.000-0.001]	180 [155-206]	188 [110-285]	7,452 [5,476-9,560]	82 [37-171]	85 [26-237]	3,393 [1,300-7,947]	43
Netherlands Antilles	0.001 [0.001-0.004]	3 [2-3]	2 [1-3]	84 [60-110]	4 [2-7]	2 [1-7]	114 [46-254]	140
New Caledonia	0.000 [0.000-0.000]	4 [4-5]	4 [2-6]	160 [116-207]	0 [0-0]	0 [0-0]	0 [0-0]	74
New Zealand	0.000 [0.000-0.000]	64 [57-72]	71 [43-105]	2,700 [2,042-3,405]	0 [0-0]	0 [0-0]	0 [0-0]	61
Nicaragua	0.002 [0.001-0.006]	137 [123-150]	88 [46-146]	4,625 [3,392-6,010]	210 [49-652]	136 [18-633]	7,111 [1,346-26,071]	178
Niger	0.007 [0.002-0.016]	810 [781-840]	1,822 [1,455-2,229]	50,167 [44,445-56,175]	5,310 [3,653-7,640]	11,937 [6,805-20,284]	328,720 [207,823-511,112]	4
Nigeria	0.014 [0.001-0.029]	6,645 [6,339-6,951]	10,282 [7,772-13,141]	335,890 [290,258-384,078]	91,011 [77,881-106,106]	140,837 [95,487-200,604]	4,600,639 [3,566,180-5,863,269]	1
Norway	0.000 [0.000-0.000]	61 [53-69]	74 [45-109]	2,674 [1,987-3,408]	0 [0-0]	0 [0-0]	0 [0-2]	57
Oman	0.000 [0.000-0.000]	49 [44-55]	31 [15-53]	1,577 [1,091-2,113]	0 [0-1]	0 [0-1]	5 [0-38]	119
Pakistan	0.000 [0.000-0.000]	4,778 [4,404-5,151]	3,672 [2,157-5,580]	175,117 [136,099-217,364]	224 [48-946]	173 [24-1,025]	8,228 [1,495-39,918]	172
Panama	0.002 [0.001-0.006]	70 [62-77]	61 [34-97]	2,666 [1,958-3,459]	146 [71-279]	128 [39-352]	5,581 [2,243-12,577]	163
Papua New Guinea	0.000 [0.000-0.000]	210 [196-224]	248 [168-343]	9,381 [7,652-11,231]	0 [0-0]	0 [0-0]	0 [0-0]	60
Paraguay	0.000 [0.000-0.000]	160 [146-174]	145 [87-219]	6,294 [4,837-7,888]	7 [1-40]	7 [1-51]	293 [33-1,828]	135
Peru	0.000 [0.000-0.000]	585 [524-646]	434 [241-683]	20,878 [15,487-26,760]	0 [0-0]	0 [0-0]	0 [0-2]	100
Philippines	0.000 [0.000-0.000]	2,385 [2,190-2,581]	2,357 [1,454-3,483]	98,487 [76,928-121,966]	0 [0-0]	0 [0-0]	0 [0-0]	79
Poland	0.000 [0.000-0.000]	417 [343-490]	360 [192-579]	14,820 [10,304-19,703]	0 [0-5]	0 [0-5]	4 [0-183]	113
Portugal	0.000 [0.000-0.001]	94 [76-111]	73 [36-123]	3,280 [2,163-4,496]	43 [16-111]	34 [8-122]	1,509 [456-4,478]	158
Puerto Rico	0.000 [0.000-0.000]	48 [41-55]	34 [17-57]	1,606 [1,101-2,176]	2 [0-9]	2 [0-9]	72 [11-337]	134
Qatar	0.002 [0.002-0.008]	22 [19-24]	16 [9-24]	682 [486-883]	52 [13-142]	37 [6-145]	1,606 [338-5,223]	161
Republic of Korea	0.000 [0.000-0.000]	481 [394-567]	419 [225-673]	18,003 [12,448-24,010]	0 [0-0]	0 [0-0]	0 [0-0]	69
Republic of Moldova	0.000 [0.000-0.000]	42 [35-50]	23 [10-41]	1,241 [825-1,711]	0 [0-0]	0 [0-0]	0 [0-2]	101
Réunion	0.000 [0.000-0.000]	15 [13-16]	13 [7-19]	543 [400-700]	0 [0-1]	0 [0-1]	0 [0-36]	103
Romania	0.000 [0.000-0.000]	220 [181-258]	175 [91-286]	7,689 [5,269-10,369]	2 [0-25]	2 [0-27]	74 [2-995]	128
Russian Federation	0.000 [0.000-0.000]	1,685 [1,409-1,961]	1,346 [729-2,154]	58,347 [40,774-77,763]	0 [0-1]	0 [0-1]	0 [0-22]	106
Rwanda	0.001 [0.000-0.003]	466 [444-488]	609 [443-799]	21,414 [18,258-24,753]	671 [450-989]	877 [449-1,621]	30,865 [18,516-50,217]	26
Saint Lucia	0.004 [0.002-0.010]	3 [3-3]	2 [1-3]	98 [67-132]	12 [6-22]	8 [2-22]	391 [148-856]	150
Saint Vincent and the Grenadines	0.004 [0.002-0.011]	2 [2-2]	1 [1-2]	57 [40-76]	6 [2-15]	4 [1-15]	205 [53-571]	142
Samoa	0.000 [0.000-0.000]	4 [4-5]	4 [3-5]	173 [143-205]	0 [0-0]	0 [0-0]	0 [0-0]	70
Sao Tome and Principe	0.008 [0.006-0.027]	5 [5-6]	5 [3-7]	209 [165-255]	43 [11-104]	41 [7-134]	1,734 [392-4,767]	143
Saudi Arabia	0.001 [0.000-0.003]	620 [561-678]	486 [265-772]	22,722 [16,832-29,156]	763 [572-1,018]	598 [270-1,158]	27,992 [17,174-43,752]	187
Senegal	0.005 [0.001-0.012]	478 [452-504]	621 [436-838]	22,239 [18,581-26,139]	2,623 [1,955-3,450]	3,407 [1,885-5,737]	122,081 [80,389-179,029]	16
Serbia	0.000 [0.000-0.001]	108 [91-125]	81 [41-134]	3,717 [2,537-5,020]	10 [1-57]	7 [1-61]	343 [33-2,276]	144
Sierra Leone	0.013 [0.004-0.032]	226 [214-238]	217 [146-300]	9,221 [7,671-10,871]	3,009 [1,914-4,485]	2,887 [1,308-5,667]	122,831 [68,638-205,016]	184
Singapore	0.000 [0.000-0.000]	50 [41-59]	56 [31-88]	2,136 [1,477-2,844]	0 [0-0]	0 [0-0]	0 [0-0]	58
Slovakia	0.000 [0.000-0.000]	59 [48-69]	54 [29-86]	2,160 [1,492-2,883]	0 [0-1]	0 [0-1]	1 [0-32]	105
Slovenia	0.000 [0.000-0.000]	20 [17-24]	19 [11-31]	761 [533-1,008]	0 [0-2]	0 [0-2]	3 [0-82]	108
Solomon Islands	0.000 [0.000-0.000]	17 [16-19]	21 [15-29]	791 [650-942]	0 [0-0]	0 [0-0]	0 [0-0]	63
Somalia	0.000 [0.000-0.000]	429 [411-446]	960 [770-1,170]	26,106 [23,133-29,223]	14 [2-76]	31 [3-201]	851 [102-5,007]	39
South Africa	0.000 [0.000-0.000]	1,043 [934-1,153]	762 [418-1,211]	36,551 [26,993-47,101]	73 [25-197]	54 [11-207]	2,572 [719-8,070]	164
Spain	0.000 [0.000-0.001]	496 [414-579]	479 [273-743]	18,748 [13,230-24,714]	154 [55-411]	149 [36-527]	5,822 [1,757-17,530]	153
Sri Lanka	0.000 [0.000-0.000]	362 [322-403]	248 [133-398]	12,198 [8,964-15,756]	2 [1-3]	1 [0-3]	58 [23-131]	131
Sudan	0.003 [0.001-0.008]	1,475 [1,388-1,562]	1,759 [1,199-2,424]	66,243 [54,634-78,641]	4,890 [3,553-6,685]	5,831 [3,071-10,371]	219,607 [139,892-336,510]	15
Suriname	0.002 [0.001-0.008]	10 [8-11]	7 [4-11]	333 [244-431]	19 [5-60]	14 [2-62]	680 [151-2,454]	154
Swaziland	0.000 [0.000-0.000]	35 [32-38]	27 [16-41]	1,282 [994-1,596]	4 [1-14]	3 [1-16]	157 [33-600]	138
Sweden	0.000 [0.000-0.000]	114 [99-129]	129 [78-193]	4,809 [3,566-6,144]	0 [0-0]	0 [0-0]	0 [0-7]	55
Switzerland	0.000 [0.000-0.001]	77 [65-90]	77 [43-121]	3,087 [2,188-4,052]	6 [1-42]	6 [0-57]	259 [20-1,910]	53
Syria	0.002 [0.000-0.004]	465 [423-507]	390 [216-613]	18,238 [13,716-23,164]	846 [567-1,291]	709 [290-1,561]	33,145 [18,365-58,952]	186
Tajikistan	0.000 [0.000-0.000]	197 [182-213]	167 [101-249]	7,506 [5,895-9,257]	0 [0-0]	0 [0-0]	0 [0-0]	90

Country/Region	SCA frequency [IQR][1]	Births 2010 [CI][2]	Births 2050 [CI][2]	Births 2010-2050 [CI][3]	Newborns with SCA 2010 [CI][4]	Newborns with SCA 2050 [CI][4]	Newborns with SCA 2010-2050 [CI][5]	Rank[6]
TFYR Macedonia	0.000 [0.000-0.002]	22 [18-26]	16 [8-27]	741 [497-1,016]	9 [2-29]	6 [1-31]	293 [59-1,152]	*141*
Thailand	0.000 [0.000-0.000]	802 [670-933]	637 [319-1,067]	28,097 [18,621-38,654]	0 [0-0]	0 [0-0]	0 [0-0]	*93*
Timor-Leste	0.000 [0.000-0.000]	46 [44-48]	66 [48-88]	2,330 [1,991-2,687]	0 [0-0]	0 [0-0]	0 [0-0]	*65*
Togo	0.010 [0.002-0.022]	197 [184-209]	194 [125-276]	8,042 [6,503-9,694]	2,037 [1,616-2,532]	2,006 [1,102-3,343]	83,321 [57,158-117,258]	170
Tonga	0.000 [0.000-0.000]	3 [3-3]	2 [2-3]	106 [88-125]	0 [0-0]	0 [0-0]	0 [0-0]	*72*
Trinidad and Tobago	0.003 [0.001-0.008]	19 [16-22]	12 [6-21]	606 [415-822]	56 [28-105]	36 [11-99]	1,771 [705-3,874]	*165*
Tunisia	0.000 [0.000-0.001]	178 [155-201]	133 [72-213]	5,949 [4,249-7,772]	84 [52-133]	63 [24-141]	2,810 [1,433-5,132]	*166*
Turkey	0.000 [0.000-0.001]	1,269 [1,112-1,426]	929 [489-1,510]	43,844 [31,264-57,622]	327 [216-586]	240 [95-621]	11,311 [6,069-23,676]	*182*
Turkmenistan	0.000 [0.000-0.000]	110 [98-122]	78 [42-124]	3,784 [2,789-4,869]	0 [0-0]	0 [0-0]	0 [0-11]	*104*
Uganda	0.007 [0.001-0.015]	1,591 [1,524-1,658]	2,512 [1,859-3,261]	82,923 [71,294-95,267]	10,877 [8,444-13,776]	17,170 [10,300-27,087]	566,896 [395,123-791,342]	5
Ukraine	0.000 [0.000-0.000]	489 [407-572]	374 [197-606]	16,569 [11,475-22,214]	0 [0-2]	0 [0-2]	2 [0-77]	*112*
United Arab Emirates	0.000 [0.000-0.000]	97 [83-112]	90 [48-144]	3,469 [2,329-4,728]	7 [2-21]	6 [1-28]	233 [52-905]	*130*
United Kingdom	0.000 [0.000-0.001]	764 [662-866]	845 [502-1,278]	32,436 [23,828-41,727]	302 [144-598]	334 [109-882]	12,816 [5,176-28,793]	*36*
United Republic of Tanzania	0.006 [0.001-0.013]	1,989 [1,899-2,080]	4,130 [3,133-5,269]	116,952 [100,662-134,301]	11,877 [9,593-14,529]	24,655 [15,830-36,811]	698,257 [508,554-938,211]	2
United States of America	0.001 [0.000-0.002]	4,351 [3,827-4,874]	5,174 [3,199-7,614]	188,369 [141,775-238,185]	2,842 [1,792-4,443]	3,379 [1,498-6,941]	123,034 [66,372-217,131]	*21*
United States Virgin Islands	0.000 [0.000-0.001]	1 [1-1]	1 [0-2]	43 [31-57]	0 [0-1]	0 [0-1]	9 [1-55]	*120*
Uruguay	0.000 [0.000-0.000]	49 [43-55]	40 [22-64]	1,792 [1,290-2,350]	1 [0-4]	1 [0-4]	28 [3-164]	*125*
Uzbekistan	0.000 [0.000-0.000]	588 [523-653]	391 [208-633]	19,657 [14,369-25,439]	0 [0-0]	0 [0-0]	0 [0-1]	*97*
Vanuatu	0.000 [0.000-0.000]	7 [7-8]	10 [7-13]	343 [280-410]	0 [0-0]	0 [0-0]	0 [0-0]	*64*
Venezuela (Bolivarian Republic of)	0.001 [0.000-0.002]	597 [535-660]	487 [266-782]	22,173 [16,218-28,844]	452 [302-678]	369 [150-804]	16,768 [9,160-29,637]	*181*
Viet Nam	0.000 [0.000-0.000]	1,434 [1,198-1,597]	958 [483-1,615]	46,132 [31,469-62,078]	0 [0-0]	0 [0-0]	0 [0-0]	*88*
Western Sahara	0.000 [0.000-0.001]	12 [11-14]	10 [6-16]	461 [344-586]	4 [1-15]	3 [0-18]	149 [23-653]	*132*
Yemen	0.000 [0.000-0.000]	969 [920-1,018]	1,302 [920-1,749]	45,683 [38,281-53,557]	86 [30-233]	115 [30-401]	4,037 [1,233-12,266]	*37*
Zambia	0.009 [0.001-0.020]	659 [632-685]	1,683 [1,354-2,049]	42,398 [37,432-47,631]	6,039 [4,927-7,367]	15,440 [10,552-22,052]	388,855 [291,626-512,562]	3
Zimbabwe	0.001 [0.000-0.003]	381 [350-412]	309 [180-473]	14,537 [11,227-18,155]	507 [315-798]	411 [162-916]	19,341 [10,077-35,203]	183
**WHO regions[6]**								
AFRO	0.007 [0.000-0.015]	32,106 [30,388-33,824]	44,589 [32,306-58,967]	1,532,888 [1,292,478-1,789,440]	237,381 [191,067-295,354]	347,674 [217,838-536,072]	11,697,397 [8,461,417-16,020,136]	/
AMRO	0.001 [0.000-0.002]	15,471 [13,658-17,284]	13,833 [7,880-21,602]	587,398 [429,357-761,868]	11,143 [6,305-19,823]	9,596 [3,503-23,899]	417,065 [195,281-862,232]	/
EMRO	0.001 [0.000-0.001]	15,831 [14,549-17,112]	15,426 [9,891-22,283]	633,872 [500,984-777,570]	10,559 [6,242-19,390]	10,791 [4,529-26,235]	433,457 [223,215-897,309]	/
EURO	0.000 [0.000-0.001]	10,947 [9,344-12,549]	9,742 [5,472-15,258]	405,806 [290,664-531,201]	1,939 [932-4,330]	1,902 [604-5,717]	75,897 [30,533-192,299]	/
SEARO	0.001 [0.000-0.003]	37,344 [33,426-41,262]	27,993 [15,384-44,575]	1,329,998 [979,394-1,718,969]	44,454 [33,696-59,338]	33,910 [15,938-64,943]	1,605,997 [1,007,529-2,501,090]	/
WPRO	0.000 [0.000-0.000]	23,353 [19,862-26,772]	17,604 [9,505-28,307]	803,894 [563,362-1,068,964]	6 [1-23]	7 [1-36]	249 [46-1,122]	/
**HbS regions[7]**								
Eurasia	0.000 [0.000-0.001]	0 [0-0]	0 [0-0]	0 [0-0]	5,130 [2,474-11,179]	4,478 [1,385-13,518]	193,796 [77,985-484,394]	/
Americas	0.001 [0.000-0.002]	0 [0-0]	0 [0-0]	0 [0-0]	11,181 [6,324-19,896]	9,628 [3,514-23,983]	418,472 [195,879-865,325]	/
Sub-Saharan Africa	0.007 [0.000-0.015]	33,356 [31,611-35,100]	46,876 [34,049-61,859]	1,604,473 [1,355,472-1,870,048]	242,187 [194,549-302,012]	353,533 [220,901-546,741]	11,916,113 [8,599,975-16,361,830]	/
Southeast Asia	0.000 [0.000-0.000]	0 [0-0]	0 [0-0]	0 [0-0]	7 [1-32]	8 [1-46]	279 [48-1,503]	/
Arab-India	0.001 [0.000-0.003]	0 [0-0]	0 [0-0]	0 [0-0]	47,264 [35,050-65,640]	36,540 [16,730-73,326]	1,713,342 [1,049,712-2,784,723]	
**World**								
**World**	**0.002 [0.000-0.005]**	**135,775 [121,853-149,622]**	**129,904 [80,863-192,085]**	**4,667,193 [4,077,536-6,685,367]**	**305,773 [238,400-398,775]**	**404,190 [242,530-657,634]**	**14,242,161 [9,923,623-20,498,521]**	**/**

[1] New output from the Bayesian geostatistical model described in Piel et al [21]. The interquartile range (IQR) corresponds to the 25% and 75% quantiles of the full posterior predictive distribution.

[2] In thousands. From the 2010 Revision of the United Nations World Population Prospects [22]. Confidence intervals (CI) based on the low- and high-fertility variants.

[3] In thousands. Sum of the projected births between 2010 and 2050 based on the 2010 Revision of the United Nations World Population Prospects [22]. Confidence intervals (CI) based on the low- and high-fertility variants.

[4] Product of [1] and [2]. The lower bounds of the CI are based on the product of the 25% quantile of SCA frequencies and the low-fertility variant birth counts. The higher bounds of the CI are based on the product of the 75% quantile of SCA frequencies and the high-fertility variant birth counts.

[5] Product of [1] and [3]. The lower bounds of the CI are based on the product of the 25% quantile of SCA frequencies and the low-fertility variant birth counts. The higher bounds of the CI are based on the product of the 75% quantile of SCA frequencies and the high-fertility variant birth counts.

[6] As defined at http://www.who.int/about/regions/en/index.html.

[7] As shown in Web Figure 8, Web Appendix 2 (p. 23) in Piel et al [21].

[8] Calculated based on the difference between the medium estimates of SCA births in 2050 and 2010. The lowest rank corresponds to the largest increase between 2010 and 2050. The lowest rank corresponds to the largest decrease over the study period. Countries not shown in Figure 3 are italicized.
