# Supplementary material for: Global Burden of Sickle Cell Anaemia in Children under Five, 2010–2050: Modelling Based on Demographics, Excess Mortality, and Interventions
Source: PLoS Med. 2013 Jul 16;10(7):e1001484. doi: 10.1371/journal.pmed.1001484 (PMC3712914; doi:10.1371/journal.pmed.1001484)
Supplement: Table S3 — Indirect economic and mortality indicators per country. (PDF) [file pmed.1001484.s004.pdf]

Table S3 Indirect economic and mortality indicators per country

Country	Income group class[#]	Newborns with SCA (bSCA)[£]		Gross Domestic Product per Capita (GDPpc)[$]		Under-five mortality (U5m)[*]	
		2010	2050	2010	2050	2010	2050
Afghanistan	Low	0	1	/	/	184	83
Albania	Medium low	29	18	7,547.35	42,956.36	19	10
Algeria	Medium high	187	135	7,603.91	17,568.47	27	12
Angola	Medium low	9,476	9,417	5,025.81	19,173.06	156	56
Argentina	Medium high	4	4	14,388.80	38,299.32	14	7
Armenia	Medium low	0	0	4,893.84	65,852.18	27	15
Aruba	/	1	1	/	/	17	9
Australia	High	6	7	34,410.35	69,263.39	5	4
Austria	High	1	1	35,166.25	57,982.78	5	3
Azerbaijan	Medium high	0	0	/	/	43	17
Bahamas	High	8	7	29,804.64	50,850.94	18	9
Bahrain	High	87	70	21,262.52	33,266.41	9	6
Bangladesh	Low	14	9	1,495.23	7,694.82	51	16
Barbados	High	5	4	18,247.88	36,601.79	14	8
Belarus	Medium high	0	0	12,317.42	116,868.45	9	7
Belgium	High	29	32	33,454.20	50,552.18	5	4
Belize	Medium low	51	43	6,566.16	21,202.02	21	14
Benin	Low	4,998	6,661	1,398.51	5,496.62	121	41
Bhutan	Medium low	0	0	5,045.25	25,228.87	52	19
Bolivia (Plurinational State of)	Medium low	0	0	4,150.46	17,976.48	54	14
Bosnia and Herzegovina	Medium high	1	1	/	/	16	12
Botswana	Medium high	4	3	11,680.66	52,655.25	46	19
Brazil	Medium high	3,152	2,296	10,033.51	26,811.87	24	10
Brunei Darussalam	High	0	0	45,718.20	28,686.74	6	4
Bulgaria	Medium high	16	12	11,522.37	46,168.84	11	8
Burkina Faso	Low	3,806	5,924	1,110.95	6,226.98	147	45
Burundi	Low	910	677	514.05	1,532.77	152	53
Cambodia	Low	0	0	1,902.60	15,548.31	69	17
Cameroon	Medium low	7,368	7,941	2,077.08	7,137.70	136	51
Canada	High	48	57	35,447.10	68,871.56	6	4
Cape Verde	Medium low	17	11	3,437.89	15,485.66	22	10
Central African Republic	Low	1,050	1,045	718.01	2,677.45	155	60
Chad	Low	2,269	2,732	1,336.70	2,079.14	195	71
Chile	Medium high	0	0	14,091.19	44,794.61	8	6
China	Medium high	0	0	6,592.59	56,428.10	24	12
China, Hong Kong SAR	High	0	0	41,820.74	77,916.93	3	3
China, Macao SAR	High	0	0	/	/	5	4
Colombia	Medium high	323	255	8,472.90	17,720.06	23	10
Comoros	Low	28	40	/	/	86	25
Congo	Medium low	1,855	2,396	/	/	104	38
Costa Rica	Medium high	61	47	10,380.62	33,021.25	11	6
Côte d'Ivoire	Medium low	3,632	4,097	1,691.80	4,374.07	107	37
Croatia	High	1	1	/	/	7	5
Cuba	Medium high	226	176	/	/	6	5
Cyprus	High	2	2	/	/	5	4
Czech Republic	High	0	0	23,763.52	71,100.98	4	3
Dem. People's Republic of Korea	/	0	0	/	/	32	13
Democratic Republic of the Congo	Low	42,065	44,663	3,811.66	4,135.73	180	75
Denmark	High	0	0	32,700.07	51,626.96	5	4
Djibouti	Medium low	0	0	2,050.78	4,255.67	104	31
Dominican Republic	Medium high	240	173	8,390.43	45,588.95	28	11
Ecuador	Medium low	0	0	/	/	23	10
Egypt	Medium low	1,176	971	5,537.72	30,222.59	25	11
El Salvador	Medium low	79	53	/	/	23	10
Equatorial Guinea	High	444	478	/	/	151	54
Eritrea	Low	10	10	/	/	62	24
Estonia	High	0	0	16,676.17	107,617.52	7	5
Ethiopia	Low	141	111	927.88	5,586.91	96	33
Fiji	Medium low	0	0	3,967.89	11,593.79	22	13
Finland	High	0	0	31,549.07	53,295.02	3	3
France	High	531	576	30,765.50	50,375.24	4	3
French Guiana	/	6	8	/	/	15	8
French Polynesia	/	0	0	/	/	10	7
Gabon	Medium high	966	1,085	13,464.66	16,008.64	64	26
Gambia	Low	471	564	1,888.45	9,723.47	93	30
Georgia	Medium low	0	0	4,644.63	65,582.36	27	13
Germany	High	87	90	32,826.69	49,025.53	4	3
Ghana	Medium low	6,019	6,855	1,439.30	5,512.84	63	26
Greece	High	202	211	24,790.71	58,063.04	5	5
Grenada	Medium high	5	3	/	/	15	8
Guadeloupe	/	13	11	/	/	8	5
Guam	/	0	0	/	/	10	6
Guatemala	Medium low	286	336	4,320.94	11,056.54	34	15
Guinea	Low	5,735	7,123	1,004.95	5,421.75	134	46
Guinea-Bissau	Low	197	247	1,045.47	2,206.68	181	70

Country	Income group class[#]	Newborns with SCA (bSCA)[£]		Gross Domestic Product per Capita (GDPpc)[$]		Under-five mortality (U5m)[*]	
		2010	2050	2010	2050	2010	2050
Guyana	Medium low	17	11	2,663.98	10,189.12	46	22
Haiti	Low	1,089	831	998.03	2,693.79	76	26
Honduras	Medium low	819	682	3,511.72	14,771.00	33	14
Hungary	High	0	0	17,146.66	56,092.60	7	5
Iceland	High	0	0	31,611.23	52,271.72	3	2
India	Medium low	43,496	33,890	3,061.75	19,552.50	65	34
Indonesia	Medium low	1	1	3,879.17	12,110.21	31	17
Iran (Islamic Republic of)	Medium high	764	506	11,212.23	37,979.38	31	14
Iraq	Medium low	750	1,095	/	/	41	15
Ireland	High	0	0	36,707.21	57,646.32	4	4
Israel	High	237	259	26,609.29	62,560.95	4	2
Italy	High	88	92	27,000.73	34,343.14	4	3
Jamaica	Medium high	135	76	/	/	26	13
Japan	High	0	0	31,418.23	63,821.38	3	3
Jordan	Medium high	145	112	5,020.75	26,238.94	22	11
Kazakhstan	Medium high	0	0	11,099.94	72,379.46	29	14
Kenya	Low	5,062	6,776	1,511.95	5,642.90	89	34
Kuwait	High	112	146	46,122.21	47,943.02	10	6
Kyrgyzstan	Low	0	0	2,031.25	16,387.50	42	23
Lao People's Democratic Republic	Medium low	0	0	2,290.03	15,210.14	46	14
Latvia	Medium high	0	0	12,827.62	96,920.52	8	5
Lebanon	Medium high	246	176	12,384.17	40,276.33	24	11
Lesotho	Medium low	0	0	1,449.25	7,523.37	89	31
Liberia	Low	621	841	/	/	107	32
Libyan Arab Jamahiriya	High	168	123	/	/	15	8
Lithuania	Medium high	0	0	15,246.39	104,906.62	9	6
Luxembourg	High	0	0	68,621.91	108,000.92	3	3
Madagascar	Low	4,121	6,066	867.01	2,625.54	58	22
Malawi	Low	2,033	3,990	830.66	3,395.86	119	54
Malaysia	Medium high	0	0	13,167.10	46,284.79	9	5
Maldives	Medium high	0	0	6,560.49	22,009.54	12	6
Mali	Low	3,664	5,203	952.99	3,953.90	173	58
Malta	High	2	2	22,989.80	52,844.37	7	5
Martinique	/	12	9	/	/	8	5
Mauritania	Medium low	492	579	2,076.54	6,736.86	106	35
Mauritius	Medium high	0	0	11,916.47	39,171.14	15	10
Mayotte	/	13	16	/	/	6	6
Mexico	Medium high	643	461	12,379.02	34,016.73	17	7
Micronesia (Fed. States of)	Medium low	0	0	/	/	38	14
Mongolia	Medium low	0	0	3,618.05	28,621.64	37	14
Montenegro	Medium high	1	0	/	/	9	6
Morocco	Medium low	40	28	4,314.75	17,889.28	31	13
Mozambique	Low	1,838	2,248	836.13	8,137.66	123	41
Myanmar	/	0	0	/	/	57	15
Namibia	Medium high	33	29	/	/	39	19
Nepal	Low	11	9	1,070.70	5,945.14	39	13
Netherlands	High	83	85	36,797.61	59,173.35	5	4
Netherlands Antilles	/	3	2	/	/	14	9
New Caledonia	/	0	0	/	/	7	6
New Zealand	High	0	0	24,555.86	61,120.21	6	5
Nicaragua	Medium low	203	136	2,512.49	11,521.66	22	10
Niger	Low	6,166	11,937	654.05	2,323.74	144	45
Nigeria	Medium low	99,310	140,837	2,137.45	9,014.65	141	49
Norway	High	0	0	48,212.93	82,286.02	4	3
Oman	High	0	0	24,984.94	51,630.74	11	6
Pakistan	Medium low	225	173	2,467.31	10,381.61	86	48
Panama	Medium high	145	128	12,376.48	55,243.48	21	9
Papua New Guinea	Medium low	0	0	2,156.76	8,562.70	58	23
Paraguay	Medium low	8	7	4,645.02	13,939.03	33	13
Peru	Medium high	0	0	8,557.18	37,349.84	28	12
Philippines	Medium low	0	0	3,568.45	22,615.21	27	15
Poland	High	0	0	17,213.92	61,043.70	7	5
Portugal	High	39	34	21,498.00	42,488.23	5	4
Puerto Rico	/	2	2	/	/	9	6
Qatar	/	44	37	74,215.82	164,189.38	10	7
Republic of Korea	High	0	0	27,394.53	101,458.06	5	4
Republic of Moldova	Medium low	0	0	2,762.85	27,243.35	19	13
Réunion	/	0	0	/	/	6	6
Romania	Medium high	2	2	10,803.57	46,832.71	15	8
Russian Federation	Medium high	0	0	13,992.92	75,488.42	16	10
Rwanda	Low	704	877	1,049.78	6,957.65	114	41
Saint Lucia	Medium high	11	8	9,189.10	20,206.48	16	10
Saint Vincent and the Grenadines	Medium high	6	4	10,176.72	40,922.99	25	12
Samoa	Medium low	0	0	/	/	24	12
Sao Tome and Principe	Medium low	44	41	/	/	69	28
Saudi Arabia	High	785	598	20,695.18	24,423.50	19	10
Senegal	Medium low	2,760	3,407	1,715.83	4,497.91	85	27

Country	Income group class#	Newborns with SCA (bSCA)£		Gross Domestic Product per Capita (GDPpc)$		Under-five mortality (U5m)*	
		2010	2050	2010	2050	2010	2050
Serbia	Medium high	9	7	/	/	13	7
Sierra Leone	Low	3,060	2,887	741.61	6,673.60	157	57
Singapore	High	0	0	51,052.37	124,595.84	2	2
Slovakia	High	0	0	20,140.98	74,216.17	7	5
Slovenia	High	0	0	/	/	4	3
Solomon Islands	Medium low	0	0	2,391.90	5,351.47	43	13
Somalia	/	16	31	/	/	162	61
South Africa	Medium high	70	54	9,335.83	28,013.88	64	25
Spain	High	144	149	27,100.71	53,498.78	4	4
Sri Lanka	Medium low	2	1	4,555.06	22,009.83	13	8
Sudan	Medium low	5,151	5,831	2,104.93	5,493.41	87	29
Suriname	Medium high	19	14	7,398.00	29,055.38	27	15
Swaziland	Medium low	4	3	4,738.22	16,828.09	92	30
Sweden	High	0	0	33,614.73	58,258.56	3	3
Switzerland	High	7	6	37,918.96	51,196.62	5	3
Syria	Medium low	871	709	4,719.75	13,939.38	16	9
Tajikistan	Low	0	0	1,938.37	14,038.45	65	33
TFYR Macedonia	Medium high	8	6	/	/	15	8
Thailand	Medium high	0	0	7,676.42	25,347.38	13	8
Timor-Leste	Medium low	0	0	/	/	76	17
Togo	Low	2,082	2,006	882.32	3,395.78	104	35
Tonga	Medium low	0	0	/	/	25	14
Trinidad and Tobago	High	51	36	23,085.10	98,182.27	31	17
Tunisia	Medium high	81	63	8,623.21	33,480.68	23	11
Turkey	Medium high	313	240	12,918.79	47,546.04	23	10
Turkmenistan	Medium low	0	0	/	/	62	31
Uganda	Low	11,923	17,170	1,151.92	6,242.35	114	43
Ukraine	Medium low	0	0	6,096.34	50,881.51	15	9
United Arab Emirates	High	6	6	44,454.37	51,043.90	8	6
United Kingdom	High	312	334	32,744.90	58,177.16	6	4
United Republic of Tanzania	Low	13,446	24,655	1,256.13	8,877.29	81	28
United States of America	High	2,919	3,379	43,137.96	64,997.27	8	6
United States Virgin Islands	/	0	0	/	/	12	8
Uruguay	Medium high	1	1	12,961.44	35,619.46	15	7
Uzbekistan	Medium low	0	0	/	/	53	29
Vanuatu	Medium low	0	0	3,990.97	14,373.01	29	11
Venezuela (Bolivarian Republic of)	Medium high	445	369	10,881.88	21,697.94	20	10
Viet Nam	Medium low	0	0	2,841.12	16,414.79	23	13
Western Sahara	/	4	3	/	/	45	14
Yemen	Medium low	91	115	2,385.48	6,612.82	57	15
Zambia	Medium low	7,015	15,440	1,387.99	5,710.07	130	44
Zimbabwe	Low	534	411	/	/	71	31

Based on the 2010 gross national incomes (GNI) per capita calculated by the World Bank (http://data.worldbank.org/indicator/NY.GNP.PCAP.CD).
£ Median estimate based on estimates published in Piel et al [21].
$ Based on the BASELINE database of the French Research Center in International Economics (http://www.cepii.fr/anglaisgraph/bdd/baseline.htm).
* Based on the 2010 Revision of the United Nations World Population Prospects [22].
